# Adaptive autophagy reprogramming in Schwann cells during peripheral demyelination

**DOI:** 10.1007/s00018-022-04683-7

**Published:** 2023-01-09

**Authors:** Young Rae Jo, Yuna Oh, Young Hee Kim, Yoon Kyung Shin, Hye Ran Kim, Hana Go, Jaekyoon Shin, Hye Ji Park, Hyongjong Koh, Jong Kuk Kim, Jung Eun Shin, Kyung Eun Lee, Hwan Tae Park

**Affiliations:** 1 Peripheral Neuropathy Research Center (PNRC), Department of Molecular Neuroscience, College of Medicine, Department of Translational Biomedical Sciences, Graduate School of Dong-A University, Dong-A University, Busan, 49201 Republic of Korea; 2 Advanced Analysis Center, Korea Institute of Science and Technology (KIST), Hwarang-ro 14-gil, Seongbuk-gu, Seoul, 02792 Republic of Korea; 3 Department of Molecular and Cellular Biology, College of Medicine, Sungkyunkwan University, Suwon-Si, 16419 Republic of Korea; 4 Department of Pharmacology, College of Medicine, Dong-A University, Busan, 49201 Republic of Korea; 5 Department of Neurology, College of Medicine, Dong-A University, Busan, 49201 Republic of Korea

**Keywords:** Autophagy, Demyelination, Demyelinating neuropathy, SQSTM1/p62, Wallerian degeneration

## Abstract

The myelin sheath is an essential structure for the rapid transmission of electrical impulses through axons, and peripheral myelination is a well-programmed postnatal process of Schwann cells (SCs), the myelin-forming peripheral glia. SCs transdifferentiate into demyelinating SCs (DSCs) to remove the myelin sheath during Wallerian degeneration after axonal injury and demyelinating neuropathies, and macrophages are responsible for the degradation of myelin under both conditions. In this study, the mechanism by which DSCs acquire the ability of myelin exocytosis was investigated. Using serial ultrastructural evaluation, we found that *autophagy-related gene 7*-dependent formation of a “secretory phagophore (SP)” and tubular phagophore was necessary for exocytosis of large myelin chambers by DSCs. DSCs seemed to utilize myelin membranes for SP formation and employed p62/sequestosome-1 (p62) as an autophagy receptor for myelin excretion. In addition, the acquisition of the myelin exocytosis ability of DSCs was associated with the decrease in canonical autolysosomal flux and was demonstrated by p62 secretion. Finally, this SC demyelination mechanism appeared to also function in inflammatory demyelinating neuropathies. Our findings show a novel autophagy-mediated myelin clearance mechanism by DSCs in response to nerve damage.

**Supplementary Information:**

The online version contains supplementary material available at 10.1007/s00018-022-04683-7.

## Introduction

Macroautophagy (autophagy) is a regulatory mechanism of the cell that degrades damaged organelles, cell membranes and proteins to refill energy sources to protect against starvation. The principal features of autophagy are the formation of phagophore membranes and their expansion to become autophagosomes, which are double membrane-bound vesicles that enclose cargo and then fuse with lysosomes [1]. Recent studies have broadened the classical concept of major autophagy functioning as an energy refuel mechanism. For example, autophagy also plays a role in the digestive removal of infectious particles and toxic aggregates [2]; moreover, cells utilize autophagy machinery for the secretion of cytokines, viruses and even intracellular organelles to meet demands for homeostasis [3]. Autophagy and canonical endo/exocytosis pathways intersect at multiple levels to control exosome secretion, endocytotic recycling and synaptic vesicle sorting [4–7]. Thus, it is now believed that autophagy is a regulated intracellular renovation process of membrane-bound organelles [8].

The myelin sheath is the multiple spiral layers of the plasma membrane around neuronal axons and is essential for the rapid conduction of electrical impulses through axons. In demyelinating polyneuropathy, the myelin sheaths generated by Schwann cells (SCs), the peripheral glia, are partially or completely degraded, resulting in detrimental sensory-motor disabilities. Myelin destruction also occurs during Wallerian degeneration (WD) after peripheral nerve injury. Rapid clearance of the degenerating bulky myelin chambers, called primary myelin ovoids [9], by SCs and macrophages are necessary for competent peripheral nerve regeneration [9, 10]. Under both conditions, SCs transdifferentiate into specialized “demyelinating SCs (DSCs)” that carry out self-myelin digestion and extracellularly discharge myelin debris for phagocytosis by macrophages [9]. DSCs were recently shown to activate autophagy to digest myelin [11–13]. Induced expression of lysosomal markers in DSCs and delayed demyelination in SC-specific *autophagy-related gene 7 (Atg7)* knockout (*Atg7*-SCKO) mice support the myelin-destructive role of SC autophagy (called myelinophagy) in WD. In contrast, the mechanism by which DSCs regulate the extracellular discharge of intracellular primary myelin ovoids during WD is unknown.

In the present study, we investigated the mechanism of myelin clearance by DSCs during WD using serial block-face scanning electron microscopy (SBF-SEM) and electron tomography. We identified very large *Atg7*-dependent perimyelin membranes which enable DSCs to simultaneously exocytose large intracellular primary myelin ovoids along with small myelinosomes. Moreover, it appears that the degenerating myelin membrane provides a platform for perimyelin phagophore expansion and that an autophagy receptor, p62/sequestosome-1 (p62) mediates myelin excretion. In addition, this novel autophagy-mediated myelin exocytosis was associated with the suppression of basal digestive autophagy with inhibition of lysosomal maturation. Thus, these findings indicate that DSCs may utilize a novel form of secretory autophagy, rather than digestive autophagy, for the clearance of myelin ovoids during peripheral demyelination.

## Materials and methods

### Experimental animals

SC-specific *Atg7* conditional KO mice were generated by crossbreeding with *P0*-cre transgenic mice (obtained from Dr. Laura Feltri) and *Atg7*^*flox/flox*^ mice (obtained from Dr. Masaki Komatsu). RFP-GFP-LC3 mice and Rosa26-*tdTomato* mice were obtained from Jackson Lab (Stock No. 027139, 007,909), and the *tdTomato* mice were crossbred with *Cnp*-Cre mice [14] to generate *Cnp*-tdTomato mice. p62 KO mice were provided by Dr. J. Shin [15]. The genotypes of the mice were determined with PCR using genomic DNA extracted from tail biopsies and the primer sequences will be provided upon request.

### Human serum sampling

Serum samples were collected from 16 acute inflammatory demyelinating polyneuropathy (AIDP) patients (9 females, 7 males) and from 20 healthy controls (14 females, 6 males) who provided consent for the participation in the study. Blood samples were centrifuged at 3000 rpm for 10 min to separate the serum (plain tube, no anticoagulant). The diagnosis of AIDP was made by the respective clinical and laboratory diagnostic criteria. All AIDP serum samples were collected by the Dong-A University Neuroimmunology Team for anti-ganglioside antibody testing with a presumptive diagnosis of immune-mediated neuropathy in collaboration with the Korean Inflammatory Neuropathy Consortium [16].

### Sciatic nerve injury

After anesthesia with 10% ketamine hydrochloride (Yuhan, 0.2 ml/100 g body weight), the left sciatic nerves of adult mice (7–8 weeks) were axotomized or crushed as previously reported [17]. For morphological analysis of degenerated nerves, the distal stumps 1 mm in length from lesion sites were discarded and the next 5 mm of distal stumps were collected at the indicated times, and the nerves were immersion-fixed overnight with 4% paraformaldehyde (PFA) in the presence or absence of 2% glutaraldehyde.

### Induction of experimental autoimmune neuritis (EAN) in rats

Lewis rats were obtained from Harlan Sprague Dawley Company and bred in our animal facility. Female rats aged 7–12 weeks and weighing 160–200 g were used. Active EAN was induced in the rats as described previously [18]. Briefly, each rat was injected in both hind footpads with an emulsion containing 100 mg SP-26, a neuritogenic peptide homologous to amino acids 53–78 of bovine myelin P2 protein (Shimadzu), and Freund’s complete adjuvant (Sigma-Aldrich, F5881; M. tuberculosis H37Ra, 5 mg/mL). Each rat was treated with 500 ng pertussis toxin (Sigma-Aldrich, P7208) on days 0 and 2 post-immunization.

### Explant cultures

Sciatic nerve explant cultures were performed as previously described [17]. Sciatic nerves from uninjured or injured adult mice were removed, and connective tissues surrounding the nerves were carefully detached in calcium/magnesium-free Hank’s buffered solution under a stereomicroscope. The sciatic nerves were then cut into small explants of ~ 3 mm in length, and then cultured for the indicated time in Dulbecco’s modified Eagle’s medium containing 10% fetal bovine serum at 37 °C with 5% CO_2_. The sciatic nerve explants were used for western blotting, or the culture medium was used for ELISA.

### Immunofluorescence (IF) staining

After fixation, the sciatic nerves were cryopreserved, embedded and sectioned using a cryostat (Leica Biosystems) at a thickness of 10 μm. After blocking with PBS containing 0.2% Triton X-100 (PBST), 5% fetal bovine serum and 5% bovine serum albumin for 1 h, the sections were incubated with primary antibodies for overnight at 4 °C and washed three times with PBST. Alexa- or Cy-conjugated secondary antibodies were treated for 2 h at room temperature. For nuclear staining, slides were incubated with Hoechst 33,342 (Thermo Fisher Scientific, 62,249) for 15 min at 37 °C. Images were taken using Zeiss Imager M2 in ApoTome II microscope (Carl Zeiss) or ImageXpress Confocal High-Content Imaging System (Molecular Devices). The number of cathepsin D-positive puncta in perinuclear area of SCs in the sciatic nerves was determined, and the mean number of cathepsin D-positive puncta per SC (*n* = 30) from three independent animals was demonstrated with the standard error of the mean.

### BODIPY-FL-pepstatin A staining

BODIPY-FL-pepstatin A for active cathepsin D staining was obtained from Thermo Fisher Scientific (P12271). The experiments were conducted in accordance with the protocol provided by the manufacturer. Briefly, the sciatic nerves from uninjured or injured mice were harvested, and then connective tissues were detached in PBS. Next, the nerves were grossly teased on 60 mm dishes and incubated for 1 h at 37 °C in Dulbecco’s modified Eagle’s medium containing 10% fetal bovine serum and 1 μM BODIPY-FL-pepstatin A. The nerves were fixed in 4% PFA for 30 min, thoroughly washed with PBS and then further teased into single fibers on glass slides. The number of BODIPY-positive perinuclear puncta was counted and reported as the mean number of puncta per SC nucleus with the standard error of the mean (*n* = 30 from three independent animals).

### Measurement of autophagic flux

Unless otherwise mentioned, 2–4 month-old RFP-GFP-LC3 mice were used in this study. The sciatic nerves of mice were fixed in 4% PFA overnight, and then, the nerves were teased to isolate a single nerve fiber on a slide. Nerves were incubated with Hoechst for nuclear staining and examined using an ApoTome II microscope. In merged IF images, the numbers of RFP(+)/GFP(−) and RFP(+)/GFP(+) puncta were determined for quantification of autolysosomes. The counting was conducted in the perinuclear and non-perinuclear regions of SCs and the number of RFP(+)/GFP(−) puncta per cell at each time point was presented as the indicator of autophagic flux. In order to measure autophagic flux, at least 40 teased nerves from three animals were randomly selected and analyzed.

### p62 ELISA

Measurement of human p62 in serum was performed with commercially available ELISA kits (Lifespan Biosciences, LS-F49427). For evaluation of p62 in rats and mice, ELISA kits purchased from Enzo Life Sciences (ADI-900–212) were used. All tests were performed in triplicate according to the manufacturer’s instructions.

### Western blot analysis

For western blot analysis, sciatic nerves were grossly dissected into the small fragments, and then, the tissue lysates were made using TissueLyser LT (Qiagen) in modified RIPA buffer containing 1% Triton X-100 in Tris–EDTA solution. Proteins were centrifuged at 9000 × g for 10 min at 4 °C, and the supernatant was collected. The RIPA lysates (10–35 μg) were separated by SDS-PAGE, and then transferred onto a nitrocellulose membrane (Amersham Biosciences). After blocking with 5% nonfat dry milk in Tris-buffered saline with Tween-20 (TBST; pH. 7.2) for 1 h at room temperature, the membranes were incubated with primary antibodies (1: 500–2000) in TBST containing 1% nonfat dry milk overnight at 4 °C. After three washes with TBST, the membranes were incubated with a horseradish peroxidase-conjugated secondary antibody for 1 h at room temperature. Chemiluminescence reactions were performed using an enhanced chemiluminescence (ECL) western blotting detection system (GE Healthcare), and then, the images were detected using a Luminogragh 3 and quantified with CS Analyzer 4 software (ATTO). Quantification was performed with the density analyzer in CS Analyzer 4, and the values were obtained from three independent experiments. The list of antibodies is shown in Table S3.

### mRNA-seq analysis and gene ontology term analysis

Total RNA was extracted from uncut and distal segments of the sciatic nerve 2 days after injury. mRNA-seq analysis was performed with two biological duplicates by Macrogen (Seoul). The libraries were prepared using a TruSeq Stranded mRNA Sample Prep kit. All libraries were sequenced by the NovaSeq 6000 for 100 bp paired-end reads by Macrogen. Functional analysis of the differentially expressed genes (DEGs) was conducted by using g:Profiler tool and DAVID version 6.8 [19]. Enrichment of biological process terms was tested for statistical significance (adjusted *P* value < 0.05). Highly enriched terms were plotted in semantic space by using REVIGO [20] to visualize dependency between the Gene Ontology (GO) terms. The gene lists of each GO term are displayed in Table S2.

### Reverse transcription polymerase chain reaction

For qPCR, 500 ng of total RNA was incubated with Ready-To-Go-Your-Prime First Strand Beads (Amersham Biosciences) and oligo-dT primers, as indicated by the manufacturer. SYBR Green PCR Master mix (Applied Biosystems, 4,367,659), cDNA and primers were added, and duplicate qPCR were performed with a QuantStudio 5 real-time PCR system (Thermo Fisher Scientific) at the Neuroscience Translation Research Solution Center. The PCR products were then analyzed by using Applied Biosystems 7500 software (v2.05), and quantification was performed with results from three independent experiments. The sequences of PCR primers are listed in Table S3.

### Transmission electron microscopy

Sciatic nerves fixed in 2% glutaraldehyde and 4% PFA were postfixed in 1% OsO4 for 2 h. After dehydration through graded alcohol and acetone, tissues were embedded in Epon and ultrathin sections were made with an ultramicrotome (LEICA). The sections were stained with 5% uranyl acetate and Reynold’s lead citrate and were examined under a Hitachi transmission electron microscope equipped with a digital camera (ES500W, GATAN).

### Sample preparation for SBF-SEM

Sample preparation was performed using an *en-bloc* staining protocol to observe the ultrastructure of sciatic nerves for SBF-SEM analysis. Briefly, the sciatic nerve tissues from wild type, *Atg7*-SCKO and p62 KO mice (*n* = 2 in each genotype) were fixed in a mixture of 4% PFA and 2.5% glutaraldehyde in cacodylate buffer, for 24 h. The fixed nerves were incubated with 2% OsO4 in cacodylate buffer for 90 min, and the solution was replaced with 1.5% potassium ferrocyanide in cacodylate buffer without washing. After 90 min, the samples were sequentially incubated in filtered 1% thiocarbohydrazide for 45 min at 40 °C, 2% OsO4 aqueous solution for 90 min and 1% uranyl acetate solution overnight at 4 °C. On the next day, the sample still in the uranyl acetate solution was warmed in a 50 °C oven for 120 min, and then, the solution was replaced by lead aspartate solution for 120 min at 50 °C. After washing with PBS, the samples were sequentially dehydrated by incubation in a graded ethanol series, acetone (2 times, 15 min), acetone/Spurr’s mixture and final pure Spurr’s resin (overnight at room temperature). The samples were incubated in fresh pure resin for another 6 h and polymerized for 48 h at 60 °C.

### SBF-SEM image acquisition and morphometry

Embedded samples were trimmed to 600 × 600 × 600 μm blocks using an ultramicrotome. Then, the trimmed samples were attached to the SBF-stub using conductive resin, and the four sides of the sample were coated with platinum to prevent charging. Single field emission SEM images were acquired using an Apreo2S SEM (Thermo Fisher Scientific), or serial EM images were acquired using an SBF-SEM (TeneoVS, FEI) with the following conditions—detector: T1 for backscattered imaging; acceleration voltage: 1.78 or 2 keV; image size: 4096 × 4096 pixels with 5 nm per pixel: working distance: 6.5 mm; dwell time: 2 μs. The thickness of the sections was 50–100 nm, and approximately 1000 sheets of serial images were obtained.

For the morphometric analysis of SBF-SEM images, the distal portion of the injured sciatic nerves from 2 wild-type, 2 SCKO and 2 p62 KO mice were used. The details of sample and object selection are described in Table S1. Briefly, 300 ~ 100 serial SEM images from randomly selected each of 8 DSCs from three independent nerves in each genotype were examined for morphometric analysis.

### Image processing for 3D reconstruction

The serial images obtained through SBF-SEM were inverted using Photoshop to change the grayscale to be similar to that of TEM. For the alignment of serial images, Align serial sections/Blend Montages of Etomo software were used, and 3D modeling was performed in IMOD software with the align.mrc file obtained through Etomo software [21].

### Electron tomography

The nerve sample prepared for SBF-SEM was sectioned with a thickness of 200 nm using an ultramicrotome and collected on formvar-coated slot grids. Grids were immersed into buffer containing 20 nm gold nanoparticles for fiducial marker labeling. Tiled images were obtained from − 70 degrees to + 70 degrees using TEM (Tecnai F20-Cryo, FEI). Image acquisition was performed with Etomo software.

### Myelin fraction purification and p62 binding assay

Dissected sciatic nerves in 20 mM Tris with 0.27 M sucrose were homogenized with a Polytron, homogenizer, and nuclear and mitochondrial fractions were removed by centrifugation at 3000×*g* for 10 min. The supernatant was layered on 0.83 M sucrose and ultracentrifuged at 82,000 × g for 40 min. The myelin fraction was separated between the 0.83 M and 0.27 M layers, and the recovered myelin fractions were homogenized in 0.27 M sucrose with a glass homogenizer and then pelleted via ultracentrifugation at 82,000×*g* for 15 min.

For the p62 binding assay, the isolated myelin sample was transferred to RIPA buffer, and the soluble lysates were bound to glutathione-agarose beads or ubiquitin binding associated domain (UBA)-p62 agarose beads (Enzo) for 24 h at 4 °C with rotation. The beads were washed three times with RIPA buffer and subjected to SDS-PAGE.

### Statistical analysis

Statistical analysis was performed using GraphPad Prism software (GraphPad). *P* values were obtained from Student’s two-tailed test, and the results were given as the means ± standard error of the means. *P* value < 0.05 was considered significant.

## Results

### Suppression of canonical autophagy in DSCs

**Fig. 1:**
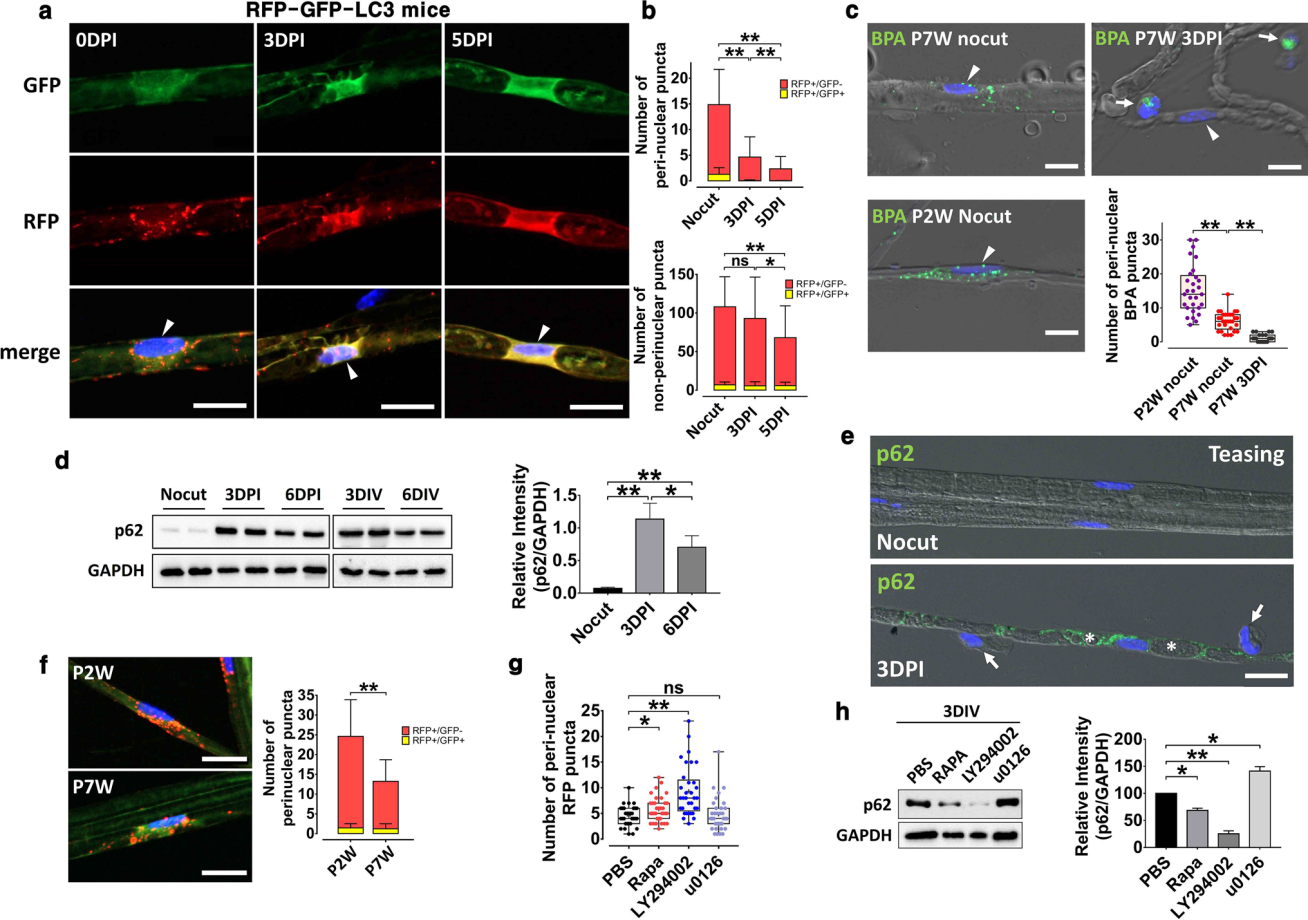
The suppression of canonical autophagy in DSCs. **a** Representative images of fluorescent autophagosomes in the perinuclear areas of DSC in the teased nerve fibers of autophagy reporter mice at adulthood. Arrowheads: SC nuclei. Scale bars in *a* = 20 μm. **b** Graphs indicate the numbers of RFP(+)/GFP(−) and RFP(+)/GFP(+) puncta in the perinuclear area and extranuclear internode. DPI: days postinjury. **c** The BODIPY FL-Pepstatin A (BPA) staining in teased sciatic nerves at postnatal 2 weeks (P2W), and before and 3 days after injury at adulthood (P7W: postnatal 7 weeks). Arrowheads: SC nuclei, Arrows macrophages. The graph indicating the number of BPA-positive puncta (green) in the perinuclear area. Scale bars in *c* = 10 μm. **d** Western blot analysis showing the levels of p62 in the sciatic nerve after injury and explant culture (DIV: days in vitro). Quantification of p62 levels in the sciatic nerves was shown in the graph. **e** IF images of teased nerve fibers showing p62 immunostaining. Asterisks: degenerating myelin chambers, Arrows: macrophages. **f** Representative images of teased nerve fibers of autophagy reporter mice at P2W and P7W. Scale bars in *e*, *f* = 20 μm. **g** Quantification of the numbers of perinuclear LC3-RFP dots in the teased nerve fiber from the sciatic nerve explant culture for 3 days from the autophagy reporter mice. **h** Western blot analysis showing the levels of p62 in the sciatic nerve explant culture with quantification. *P < 0.05, **P < 0.01, *ns* non-significant

Expansion of the initial phagophore to form autophagosomes depends on ATG8 (LC3) conjugation to phosphatidylethanolamine (called LC3II conversion) mediated by the ATG5/7–ATG12 complex [22]. According to previous studies on autophagy activation in SCs after nerve injury, an increase in the level of the LC3II form can be used as an indicator of activation [11, 12]. However, an increase in the LC3II form can be caused by the inhibition of autophagic flux; and the digestion of cargo by autolysosomal fusion [23]. Thus, we tried to assess autophagic flux in DSCs using an autophagy flux reporter mouse line, i.e., the RFP-GFP-LC3 line [24]. Since GFP fluorescence is quenched under acidic conditions (lysosomes) while RFP retains its fluorescence, an increase in the number of RFP(+)/GFP(-) LC3 puncta (RFP(+)/GFP(−) puncta) indicates an increase in autophagic flux. Teased uncut sciatic nerve preparations from adult RFP-GFP-LC3 mice (7–8 weeks old) revealed higher numbers of RFP(+)/GFP(−) puncta compared to RFP(+)/GFP(+) puncta in the perinuclear SC cytoplasm and internodal area (non-perinuclear region), suggesting the presence of basal autophagic flux (Fig. 1a). The number of RFP(+)/GFP(−) puncta in the perinuclear area of SCs was significantly decreased by 3 days postinjury (3 DPI) and was further decreased at 5 DPI (Fig. 1a, b). The mean number of RFP(+)/GFP(−) puncta in the internode was also decreased with time after injury (Fig. 1b). The decrease in RFP(+)/GFP(−) puncta in DSCs was accompanied by a general increase in green/red fluorescence, but not by an increase in RFP(+)/GFP(+) puncta, in DSCs, indicating a potential suppression of the formation of autophagosomes and autolysosomes.

To determine the negative regulation of autolysosomes in DSCs, we examined active lysosome staining in DSCs. There was a significant reduction in the number of cathepsin D-positive puncta in DSCs at 3 DPI compared to SCs in uncut nerves (Fig. S1a). Moreover, staining with BODIPY-FL-pepstatin A (BODIPY-FL), a marker of active lysosomal pepstatin A [25], also showed a dramatic decrease in the number of BODIPY-FL-positive puncta in DSCs at 3 DPI compared to the number in uncut control SCs (Fig. 1c). In addition, we examined the expression of p62, which is known to be destroyed by autolysosomes [26], in DSCs. We observed substantial increases in p62 expression in the injured sciatic nerves 3 days after injury using western blot analysis, and the accumulation of p62 was slightly reduced 6 days after injury (Fig. 1d). The protein levels of p62 were also increased in 3 and 6 days in vitro (DIV) sciatic nerve explant cultures (Fig. 1d) [12]. IF staining revealed a strong induction of perimyelin p62 staining in DSCs, but not in infiltrated leukocytes (Fig. 1e, Fig. S1b). We examined potential transcriptional induction of p62 mRNA in the injured nerves; however, mRNA-Seq analysis of the sciatic nerves after nerve injury did not reveal a significant increase in the upregulation of p62 mRNA expression (Fig. S1c), indicating that the accumulation of p62 might be attributed to lysosomal inhibition in DSCs.

Since our results indicating autolysosomal downregulation in DSCs after injury were unexpected, we next thought to validate the autophagy reporter mouse and BODIPY-FL staining results by examining the autolysosomal activity in actively myelinating SCs during postnatal development in which there are more lysosomes than in adult SCs [27]. The number of RFP(+)/GFP(−) puncta in SCs was higher in 2 week-old RFP-GFP-LC3 mice than that in adult mice (Fig. 1f). In addition, the number of BODIPY-FL puncta in SCs from 2-week-old mice was higher than that in adult SCs (Fig. 1c). Thus, the expression of lysosomes in actively myelinating SCs, adult SCs and DSCs consistently changed with the levels of autophagic flux in those cells, and the suppression of autophagic flux was considered as a specific response of SCs after nerve injury.

Because the PI3K-mTORC1 axis regulates not only autolysosome biogenesis but also SC demyelination [22, 28], we considered the involvement of this axis in the suppression of autophagic flux in DSCs. Using sciatic nerve explant cultures, we first examined whether mTORC1 activity is regulated by the PI3K or the extracellular signal regulated kinase (ERK, an SC transdifferentiation regulator [29]) pathway in DSCs and found that the inhibition of PI3K by LY294002, but not of ERK by U0126, completely suppressed the induction of pS6, an indicator of mTORC1 activity, in the sciatic nerve explant cultures, indicating that the PI3K-mTORC1 axis was activated in DSCs (Fig. S1d). Using sciatic nerve explant cultures from RFP-GFP-LC3 mice, we found that LY294002, but not U0126, very significantly prevented the reduction in autophagic flux in DSCs at 3 DIV (Fig. 1g). In addition, treatment with rapamycin, an mTORC1 inhibitor, also mildly inhibited the autophagy flux suppression in DSCs. As expected, the accumulation of p62 in DSCs in the sciatic nerve explants was also suppressed by LY294002 and rapamycin according to western blot analysis (Fig. 1h), suggesting that the PI3K-mTORC1 pathway is involved in autophagy flux inhibition in DSCs after nerve injury.

### Alteration of gene expression involved in lysosome and vesicular transport in DSCs

**Fig. 2:**
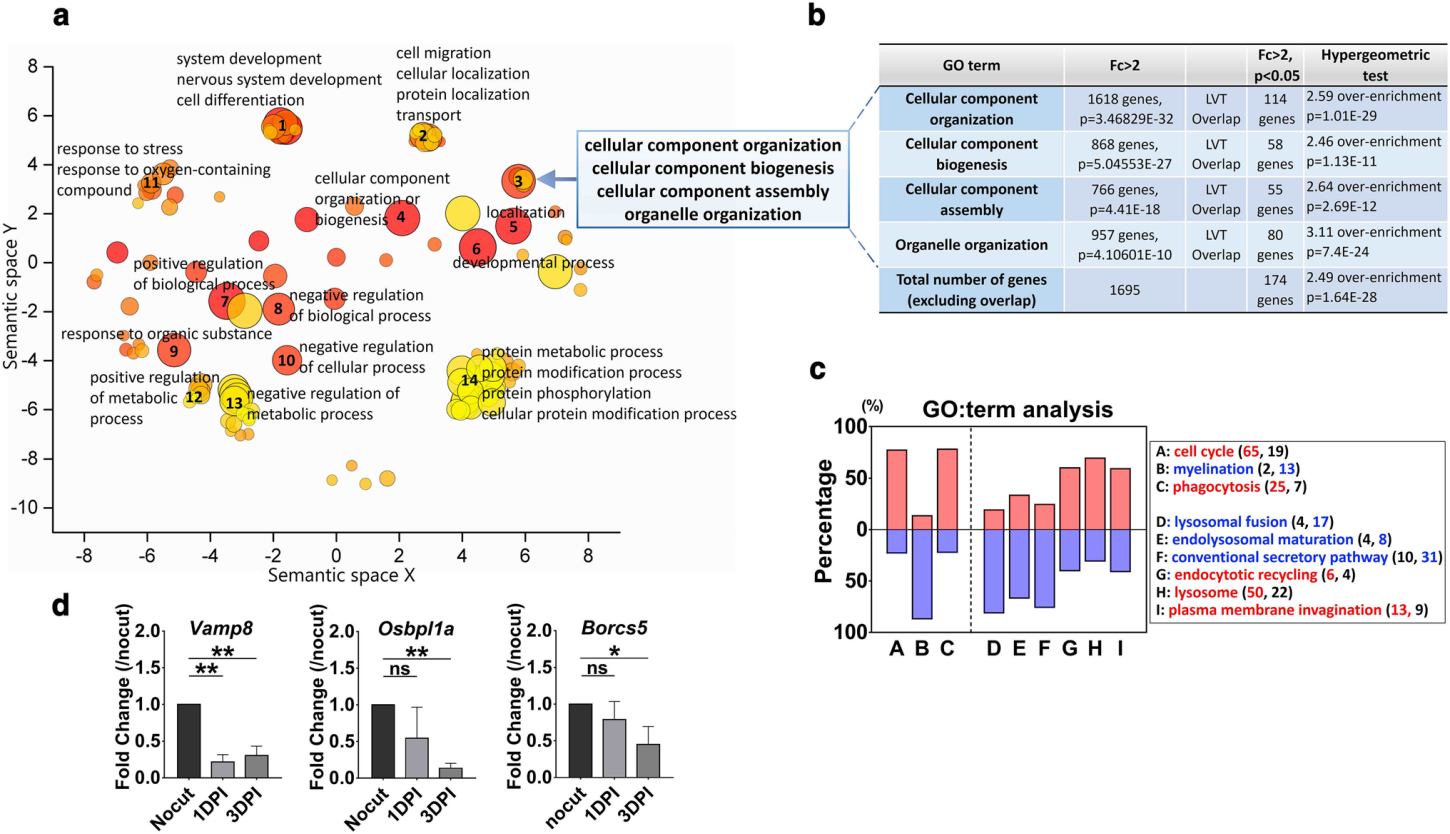
Transcriptional downregulation of autolysosomal maturation in DSCs. **a** Biological pathways of DEGs identified by DAVID GO analysis were visualized using REVIGO. The circle diameter reflects the − log10-*P* value, and colors indicate the fold enrichment scores. Horizontal and vertical axes of the plot represent semantic space (SimRel vector space); the circles closeness on the plot reflects their semantic similarity between the GO terms. **b** Overlap of genes (FC > 2, *P* < 0.05) in each GO term of “cellular compartment (blue square in *A*)” and lysosomal and vesicular transport (LVT). **c** The percentages of up (red) or downregulated LVT genes. A-C: known injury responses of SCs. D-I: arbitrary terms representing the function of 6 groups from LVT genes. **d** RT-qPCR showing the expression levels of *Borcs5, Osbpl1a* and *Vamp8* mRNAs. Mean values from three independent experiments. *P < 0.05, **P < 0.01, *ns* non-significant

To determine whether the changes in the transcriptome after nerve injury underlie the dramatic alteration of autolysosome in DSCs, we performed mRNA-Seq analysis of the sciatic nerves after nerve injury. To exclude the potential contribution of contamination of macrophage infiltration in the injured nerves, we used the distal stump of the injured sciatic nerves at 2 days after injury when macrophage infiltration had not yet begun. We identified 6397 differentially expressed genes (DEGs) in 2 day cut sciatic nerves compared to uncut nerves (fold change (FC) > 2, raw *P* < 0.05, Fig. S2a). We analyzed the biological functions of DEGs in DSCs using GO term analysis. Gene expression related to lipid synthesis, cell cycle regulation, inflammation and phagocytosis was highly enriched (adj. *P* < 0.05, Fig. S2b, c), indicating a correlation with previously known SC responses to nerve injury [13, 30]. Injury specifically regulated the gene expression in biological processes related to the assembly and organization of “cellular components” (1695 genes among 6397 genes) in DSCs (Fig. 2a, b, and Table S2). Peripheral nerve injury induces autophagy activation in sensory neurons of the dorsal root ganglion [31], and thus we analyzed GO terms of the DEGs in injured sensory neurons based on a published DEG database [32] to determine the specificity of the enrichment of “cellular component” in DSCs. We could not find enrichment of the “cellular component” in sensory neuron DEGs following injury (Fig. S2d and Table S2), indicating that the transcriptional enrichment of “cellular component” was a specific injury response of SCs, but not of sensory neurons that indicated autolysosome activation after injury.

Since the inhibition of canonical digestive autophagy potentially encompasses the alteration in cellular lysosomal and vesicular transport (LVT) systems, we compared the shared transcriptomic data between the “cellular components (1696 genes)” and LVT systems in DSCs (18 related GO terms, Table S2). A total of 115 genes out of 174 LVT genes in DSCs overlapped with the 1695 genes in DSCs (2.5 fold overenrichment than a hit by chance, *P* < 0.01, hypergeometric test, Fig. 2b and Table S2), while only 27 genes out of the 174 VLT genes were found as DEGs in the dorsal root ganglion after injury (1.63 fold under-enrichment compared with a hit by chance, *P* < 0.01, hypergeometric test, Table S2). We clustered 18 LVT-related GO terms into 6 groups based on functional similarity. The analysis of genes in each of the 6 groups (FC > 2) indicated that 81, 76 and 67% of LVT genes involved in lysosomal fusion, conventional exocytosis and endolysosomal maturation, respectively, were downregulated (Fig. 2c). We also examined the expression of 3 genes related to autolysosomal fusion based on the literature, and confirmed the downregulation of *BLOC-1 related complex subunit 5* (*Borcs5*)*, vesicle associated membrane protein 8* (*Vamp8*) and *oxysterol binding protein-like 1A* (*Osbpl1a*) in the sciatic nerves after axotomy using RT-qPCR analysis (Fig. 2d). These findings further indicated that the injury-induced suppression of the autolysosomal process was associated with correlated gene expression changes in DSCs during WD.

### SBF-SEM revealed a novel myelin exocytosis process in DSCs

**Fig. 3:**
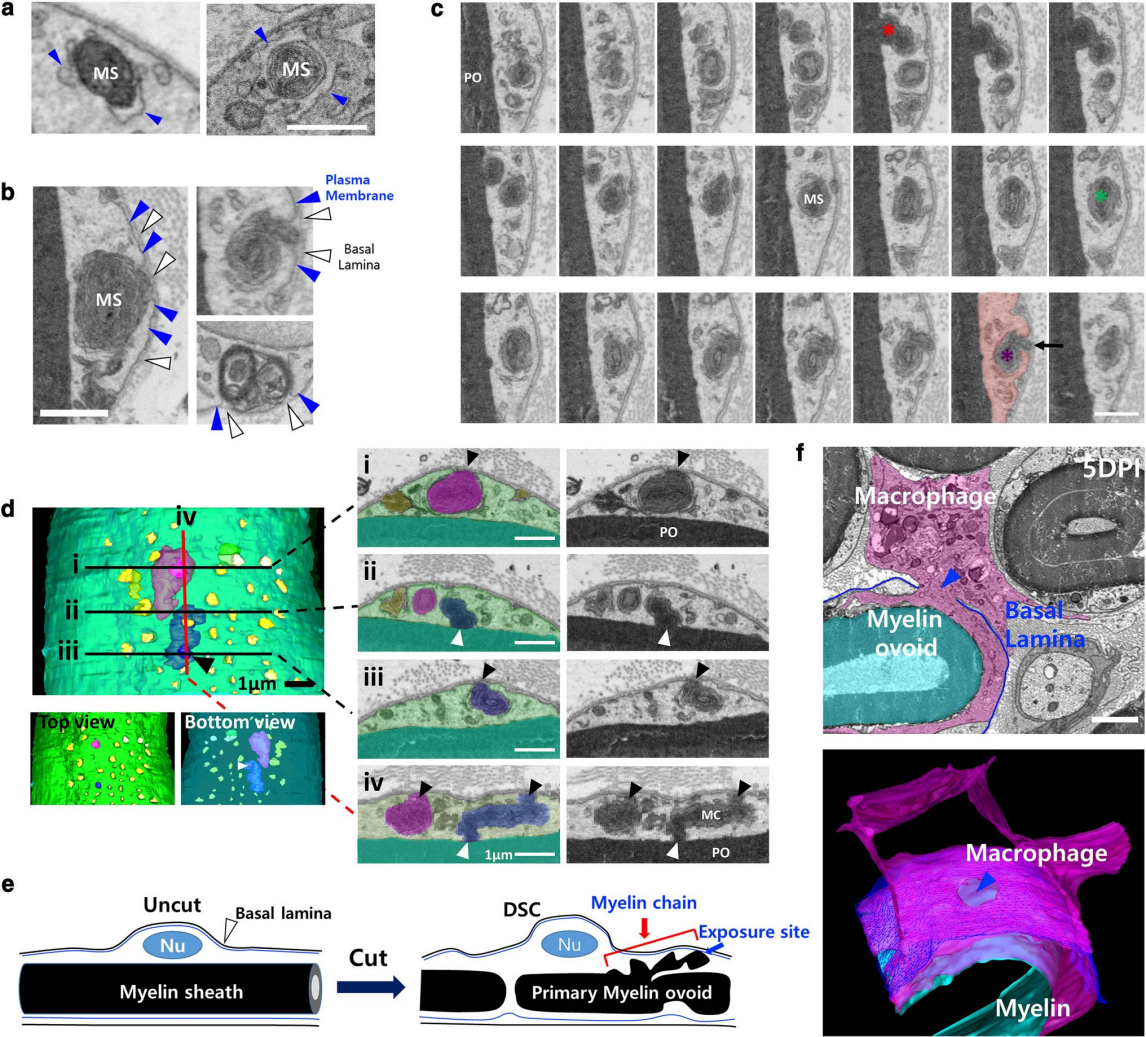
SBF-SEM images showing myelin exocytosis in DSCs. **a** SBF-SEM images of a myelinosome (MS) that was partially or completely encircled by phagophores (arrowheads) in a single section. **b** SBF-SEM images of exocytosed myelinosomes (MS) directly exposed to the basal lamina (white arrowheads). **c** SBF-SEM images showing that a myelinosome (green asterisk) was connected to the primary ovoid (PO) and also exposed to the basal lamina (purple asterisk, black arrow) on the other side. Scale bars in **a–c** = 0.5 μm. **d** Three-dimensional reconstruction showing the exposure of myelinosomes to the basal lamina (black arrowhead) with the connection of the myelinosomes to the primary ovoid (PO, white arrowhead). Top view shows the openings of intracellular myelin (arrows, magenta and blue) to the exterior. Image “iv” is a longitudinally reconstructed image of the serial cross sections through “i-iii”. *MC* myelin chain. The scale bars = 1 μm. **e** Schematic drawing of a DSC showing the primary myelin ovoid and myelin chain. **f** Serial SEM images and three-dimensional reconstruction showing a myelin-destructing macrophage (magenta color) within the endoneurial tube at 5 DPI. The scale bars = 2 μm

How does autophagy contribute to demyelination with canonical autophagy inhibition in DSCs during WD? It has been shown that the rejection of degenerating myelin by DSCs was dependent on SC autophagy [33]. To explore the mechanism, we examined the ultrastructure of DSCs carrying out myelin clearance in the sciatic nerve of wild type and *Atg7*-SCKO mice using SBF-SEM, which allows examination of 400 ~ 1036 serial SEM images (Methods, Table S1), although at a resolution lower than that achieved by TEM. At 3 DPI, a myelin debris was often enclosed in an autophagosome-like membrane in DSC cytoplasm in a single section of SBF-SEM (Fig. 3a). However, typical electron-dense lysosomes, which indicate the induction of autolysosomes and can be clearly observed in SCs at 2 weeks postnatally [27], were not found in adult DSCs by SBF-SEM at 3 DPI (Fig. S3a). Interestingly, 75.5% of the 89 myelinosomes in DSCs, each of which was seen as an isolated intracellular myelin sphere in a single section, were found to be directly exposed to the basal lamina of the endoneurium in serial SEM images (Fig. 3b, c and Fig. S3d). The secreted myelinosome, which remained within the endoneurial tube, was also clearly seen on TEM images (Fig. S3b), and a part of the secreted myelinosome was released into the extracellular space beyond the endoneurial basal lamina (Fig. S3c). Moreover, serial SEM imaging revealed that 78.7% of the 89 myelinosomes within DSCs, which appeared to be separated from primary myelin ovoids in a single SEM section, were connected to the primary ovoid by a chain of myelinosomes at 3 DPI (Fig. 3c-e). Thus, there was a chain-like myelinosome connection from the intracellular primary ovoid to the plasma membrane, even though it looked isolated in a single section (Fig. 3c, d).

The pore in the plasma membrane of DSCs, through which myelin was exposed extracellularly (Fig. 3d (top view), e), might provide an entry point for invading macrophages, allowing phagocytosis of primary intracellular ovoids. SBF-SEM images of sciatic nerves at 5 DPI showed that the surface area of myelin exposed to the outside of DSCs (the initial pore) further increased and that the myelin ovoids were intimately contacted by intratubal macrophages (Fig. 3f), suggesting that there might be an increase in the myelin exposure area of DSCs with increasing time.

### *Atg7*-dependent perimyelin phagophore expansion in DSCs

**Fig. 4:**
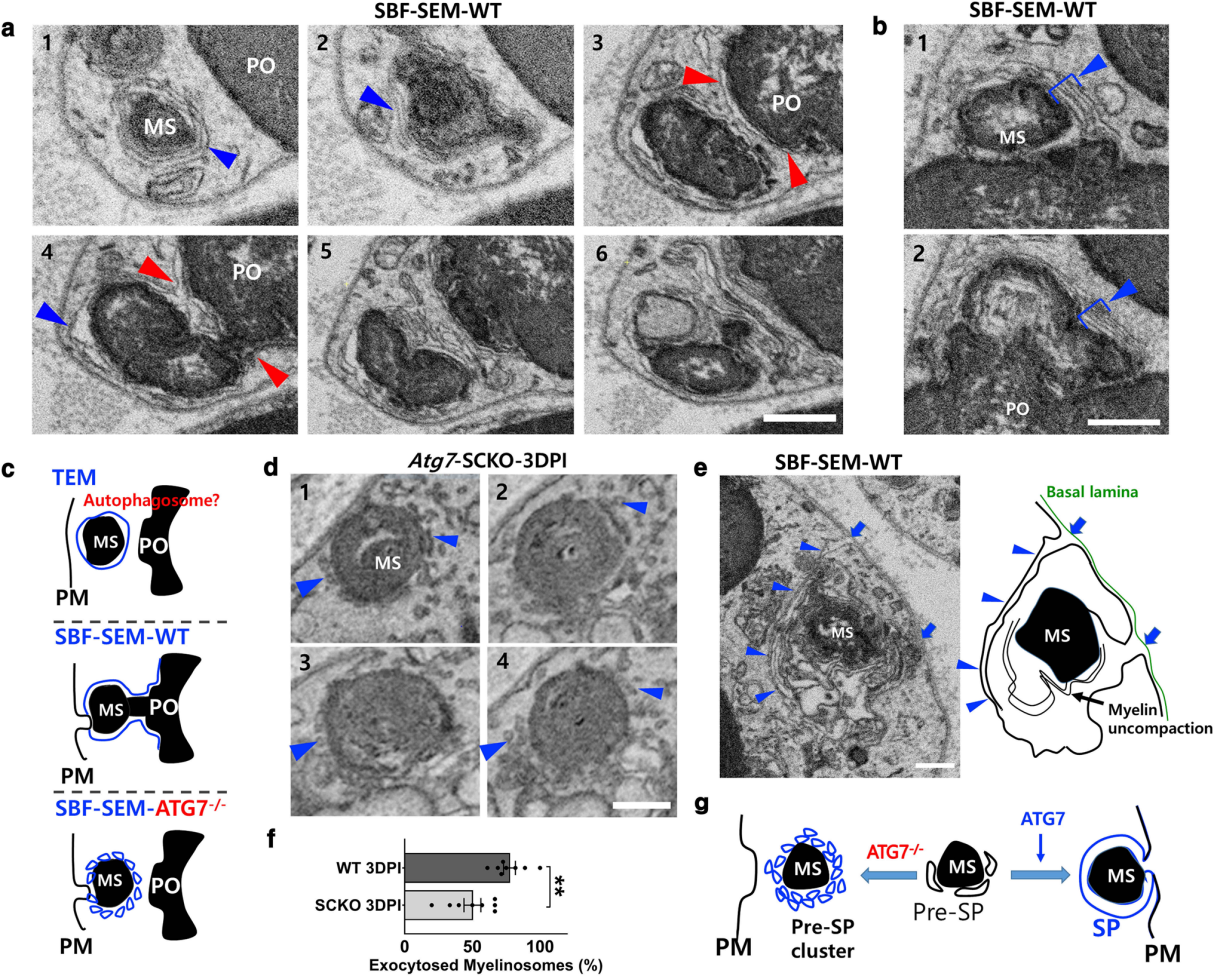
*Atg7*-dependent secretory phagophore (SP) formation in DSCs. **a** Serial SEM images of a DSC from WT mice at 3 DPI showing the connection of myelin-enclosing membranes (blue arrowheads) and the membrane covering of the primary ovoid (red arrowheads). PO: the primary ovoid. **b** SBF-SEM images showing uncompacted degenerating myelin lamellae-like membranes (blue bars with arrowheads) around a myelinosome (MS). Scale bars in *a* and *b* = 0.5 μm. **c** Three potential modes of myelin clearance by DSC. *PM* plasma membrane. **d** Serial SEM images showing multiple vesicles around a myelinosome (blue arrowheads) in *Atg7*-SCKO DSC. **e** SBF-SEM image showing the connection of outermost perimyelin membrane around a myelinosome (blue arrowheads) with plasma membrane (PM) invagination (blue arrows). Scale bars in *d*-*e* = 0.2 μm. **f** Quantification of exocytosed myelinosomes within endoneurial tube in WT and *Atg7*-SCKO mice at 3 DPI. Each dot indicates the percentage of exocytosed myelinosome in each DSC. **P < 0.01. **g** The concept of pre-SP and SP. *PM* plasma membrane

We next examined serial ultrastructures of perimyelin membranes in DSCs using SBF-SEM to determine how myelinosomes are connected to the large myelin ovoids. As shown in Fig. 3a, even though an autophagosome appeared to completely encircle the cargo, i.e., a myelinosome, in a single SEM image, it was continuous with membrane coverings of both the myelin chain and the primary myelin ovoid (Fig. 4a and Video S1), indicating that complete autophagosome closure around a myelinosome might not be feasible for this continuous structure (Fig. 4b, c). Moreover, the autophagosome-like perimyelin membranes (APPM) around myelinosomes were often indistinguishable from uncompacted degenerating myelin lamellae in DSCs, in certain single SEM sections (Fig. 4a, b and Video S1).

In contrast to the APPMs in WT DSCs, myelinosomes were covered by small ellipsoidal vesicles (~ 30 nm diameter) in the *Atg7*-SCKO mice (Fig. 4d and Fig. S4a). In *Atg7*-SCKO mice, 123 vesicular clusters were observed in 54 DSCs, whereas only 4 vesicular clusters were found in 46 WT DSCs (Table S1c). Electron tomographic imaging indicated that the small vesicular structures in *Atg7*-SCKO mice were not tubules or ER (Video S2). SBF-SEM analysis revealed that the small vesicles stuck together forming clusters of various sizes, and that none of the small vesicles within the cluster elongated further in *Atg7*-SCKO DSCs (Fig. 4d, Fig. S4a and Video S3). In addition, the cluster of small vesicles attached to the myelin was positioned around the myelin with a perimyelin-onion ring-like pattern (Fig. 4d, Fig. S4a and Video S3). Overall, these findings suggest that the small perimyelin vesicles accumulated in *Atg7*-SCKO DSCs are unexpanded small phagophores and that generation of the APPM seen in WT DSCs is an *Atg7*-dependent process (Fig. 4c).

### *Atg7*-dependent perimyelin membranes are in part required for myelin exocytosis in DSCs

We next asked whether the generation of the *Atg7*-dependent APPMs is necessary for the connection of myelinosomes to the primary ovoid. We counted the connections between isolated myelinosomes and the primary ovoid in serial SEM images of DSCs and found that 78.7% of 89 myelinosomes in WT DSCs and 54% of 126 myelinosomes in *Atg7*-SCKO DSCs were connected to the primary ovoid, respectively (Table S1b). These findings suggest that the *Atg7*-dependent APPM is required for the myelinosome connection to the primary ovoid (Fig. 4c).

As shown in Fig. 4e and Video S1, we frequently observed connections between the APPMs and the plasma membrane of WT DSCs that would lead to myelin exocytosis. To determine whether *Atg7* is required for myelin exocytosis, we compared the number of exocytosed myelinosomes (but enclosed within the endoneurial basal lamina, Table S1a) between WT and *Atg7*-SCKO mice at 3 DPI. This analysis revealed a significant decrease in exocytosed myelinosomes in *Atg7*-SCKO compared with WT DSCs (Fig. 4f). Together, these data indicate that *Atg7*-dependent phagophore expansion contributes to myelin exocytosis via fusion with the plasma membrane during WD, and thus, we introduced the concepts of “secretory phagophore (SP)” and pre-SP (Fig. 4g) to explain autophagy-dependent myelin exocytosis.

### Uncompacted degenerating myelin might provide a platform for the generation of the SP

**Fig. 5:**
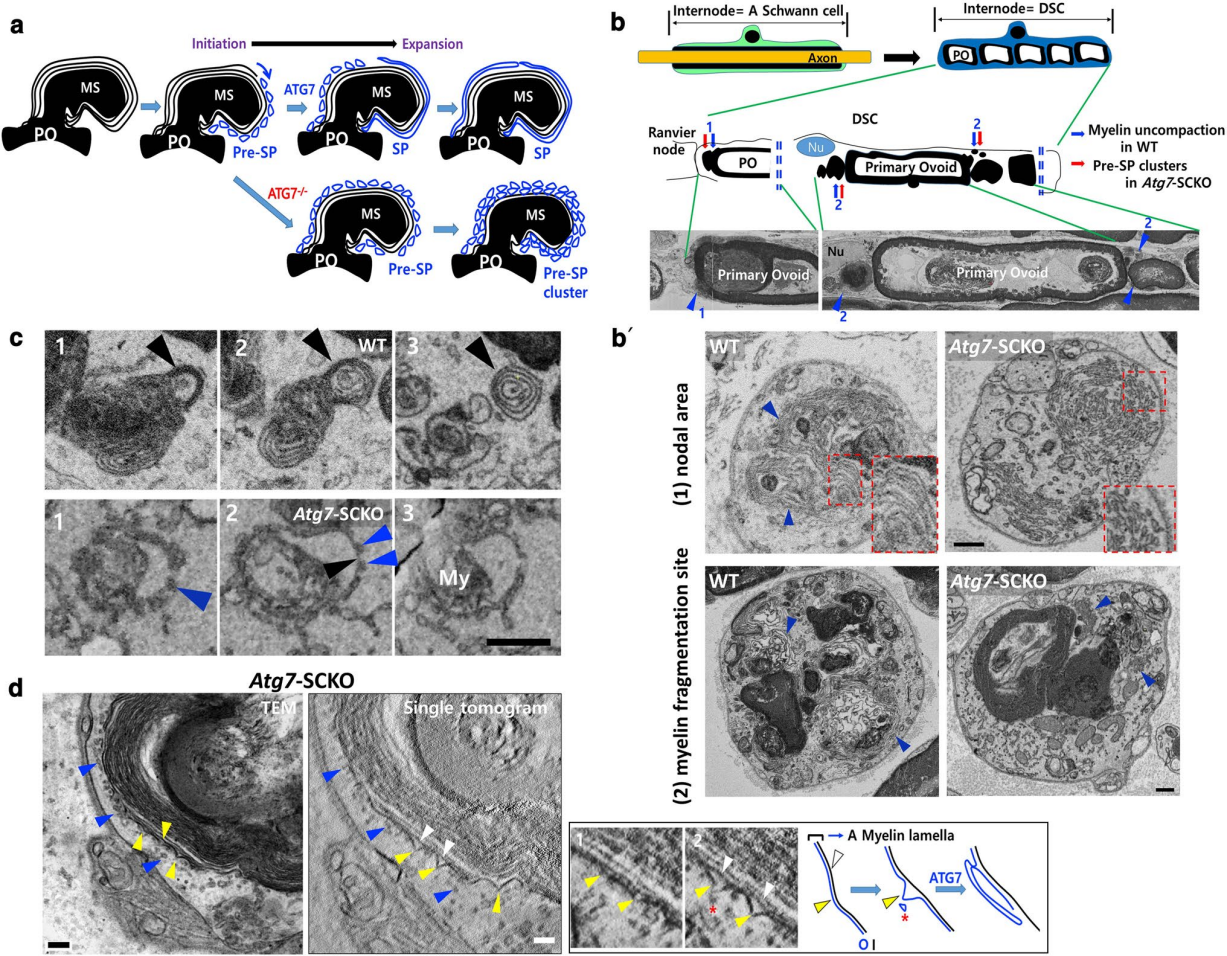
Myelin lamella as the platform for *Atg7*-dependent secretory phagophore formation in DSCs. **a** Hypothesis on the origin of SP in DSC. **b** Schematic drawing showing a process of myelin fragmentation within DSC during WD. Longitudinal SBF-SEM image was reconstructed from several hundred cross images of SBF-SEM. Nu: nucleus. 1, 2 myelin fragmentation sites. **b** Comparison of myelin membrane structures in myelin fragmentation sites (nodal area (1) and the ends of a primary ovoid (2)) of WT and *Atg7*-SCKO DSC. Red boxes in the corner: enlarged areas. Blue arrowheads: perimyelin membranes and pre-SP. **c** Comparison of myelin membranes of small myelin debris between WT and *Atg7*-SCKO DSCs. Black arrowheads: myelin membranes, blue arrowheads: pre-SP. **d** Electron tomography showing endocytotic invagination of the outer leaflet (O, yellow arrowheads) of outermost myelin lamella in *Atg7*-SCKO DSC. Inner leaflet (I, white arrowheads) was separated from the outer leaflet in the invagination areas. Blue arrowheads and red asterisk: pre-SP. The box shows a hypothetical drawing of ATG7-dependent phagophore expansion from the outer leaflet of a myelin lamella. Scale bars = 0.2 μm

We observed multiple layers of pre-SP surrounding myelinosomes in *Atg7*-SCKO DSCs (Fig. 4d and Fig. S4a). Considering that the plasma membrane is a source of autophagosomes in cells [34], we hypothesized that the outer layers of the myelin lamellae of myelin ovoids or myelinosomes (which are designated as uncompacted degenerating myelin lamellae that are distinct from the normal uncompacted region of the myelin sheath, such as the Schmidt-Lanterman incisure (SLI)) are a source of the SP (Fig. 5a). To verify this, we first compared the regional characteristics of uncompacted degenerating myelins of injured WT DSCs to those of the pre-SP cluster in *Atg7*-SCKO DSCs using SBF-SEM. Uncompacted degenerating myelin was principally observed in myelin fragmentation sites during demyelination; these sites are perinodal areas and the area between the primary ovoids in the internode of WT DSCs (regions 1 and 2 in Fig. 5b**,** see also Video S4). Interestingly, the multiple layers of uncompacted degenerating myelin at the end of the primary ovoid seen in injured WT DSCs were completely lost in *Atg7*-SCKO DSCs and replaced by multiple layers of pre-SP (Fig. 5b and Video S5). We counted the number of myelin fragmentation sites containing pre-SP cluster in WT and *Atg7*-SCKO DSCs and found that 14 out of 20 myelin fragmentation sites in *Atg7*-SCKO DSCs had pre-SP clusters, whereas there were no pre-SP clusters from 16 myelin fragmentation sites in WT DSCs. These observations suggest that the SP might be generated from the uncompacted degenerating myelin lamellae.

Three additional findings support our hypothesis of the origin of the SP. First, we found myelin membranes in a transitory state between the lamella and pre-SP in *Atg7*-SCKO DSCs. In serial SEM, it was observed that “multiple pre-SPs with one layer of membrane” changed to “multiple pre-SP without any membrane” in the next section (Fig. 5c, Fig. S5a, b). Second, there were many myelin debris with 1–2 layers of myelin lamellae surrounded by multiple layers of pre-SP clusters in *Atg7*-SCKO DSCs (24 clusters from 8 DSCs, Table S1d) (Fig. 5c and Fig. S5c), while these structures were never seen in WT DSCs. These two findings might indicate that outer myelin lamellae were used up for the continuous generation of the pre-SP, as there were only a few layers or no layer left inside the pre-SP clusters in *Atg7*-SCKO DSCs. Finally, we examined the pre-SP along the outermost myelin membrane in the *Atg7*-SCKO DSCs using electron tomography. Interestingly, the outer leaflet of the outermost myelin lamella showed intermittent conical endocytotic pit-like depressions toward the cytoplasm, separating it from the inner leaflet of the lamella. These pits were regionally associated with the pre-SP, suggesting that the outer leaflet of the outermost myelin lamella might contribute to pre-SP formation (Fig. 5d and Video S2).

The Schmidt–Lanterman incisure (SLI) in uncut nerves is the noncompacted region where myelin fragmentation occurs during WD, thereby producing the primary myelin ovoid [9]. IF staining for LC3 and WIPI-1, which interacts with phosphatidylinositol-3 phosphate to recruit the initial autophagy machinery required for LC3 conjugation [35], showed that these proteins were highly localized in the fragmentation site close to the damaging SLI in WT DSCs (Fig. S5d, e), supporting our hypothesis on the origin of the SP.

### *Atg7*-dependent tubular phagophores from the plasma membrane for myelin exocytosis in DSCs

**Fig. 6:**
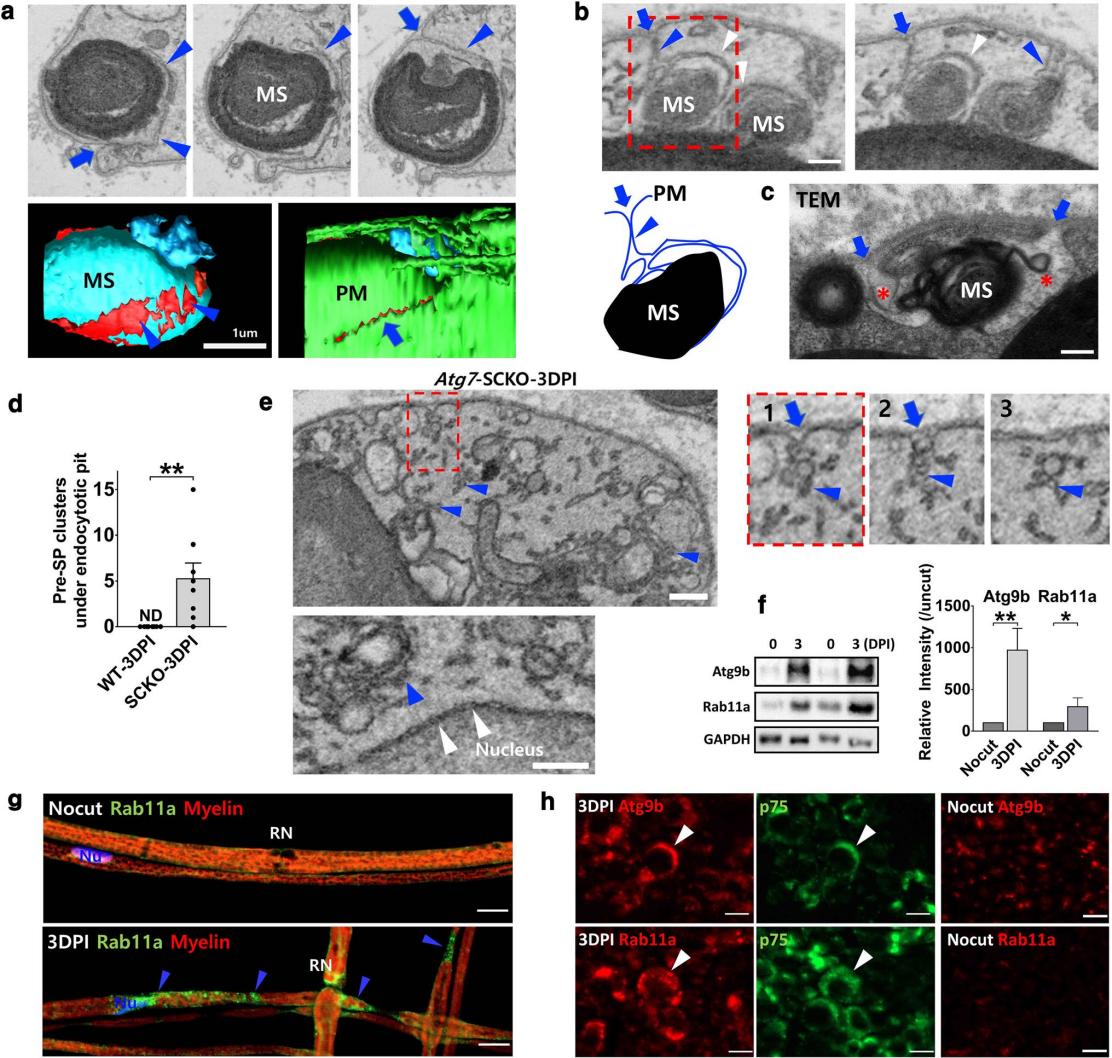
*Atg7*-dependent tubular phagophores in DSCs. a SBF-SEM images showing a tubulovesicular invagination from the plasma membrane (arrowheads) in WT DSC. Three dimensional reconstruction of the SBF-SEM images showing large area of the tubulovesicular structure (red area) and the exposure of myelin to the outside of the SC (blue arrow). PM (green): plasma membrane. Scale bar = 1 μm. **b, c** SBF-SEM (**b**) and TEM (**c**) images showing the connection of tubular phagophores (blue arrowheads) and perimyelin membrane (white arrowheads). Blue arrow: opening to the outside of DSC. Asterisk in TEM image: tubulovesicular invagination. **d** Quantification of the numbers of pre-SP cluster under endocytotic pit in WT and *Atg7*-SCKO DSC. Each dot indicates the number in each DSC. **P < 0.01. **e** SBF-SEM images showing the localization of pre-SP under the endocytotic pit (red box), but not along the nuclear membrane (white arrowheads). Scale bars in **b–e** = 0.2 μm. **f** Western blot analysis showing the levels of Atg9b and Rab11a in the sciatic nerve after nerve injury with quantification. *P < 0.05, **P < 0.01, *ns* non-significant. **g, h** Representative IF images of Rab11a and Atg9b (arrowheads) in the teased nerve fibers (**g**) and cross sections (**h**) of the sciatic nerves at 3 DPI. Scale bar = 10 μm

It was previously reported that a dramatic increase in the expression of endocytotic markers was related to demyelination of DSCs during WD [13]. Since the autophagy pathway interacts with the recycling endosome pathway to form tubular phagophores [36], we examined the potential *Atg7*-dependent endocytotic structure of the plasma membrane in DSCs at 3 DPI using SBF-SEM. We found tubular invaginations of the plasma membrane in WT DSCs, and three-dimensional reconstruction revealed that the area of the invaginated structures ultimately became enlarged to enclose intracellular myelin of various sizes (Fig. 6a). The tubular invaginations seemed to be elongated to meet the APPM for myelin exocytosis (Fig. 6b and Video S1). We also found similar invaginated tubulovesicular structures around myelin being exocytosed under TEM (Fig. 6c). In contrast, the pre-SP clusters observed in *Atg7*-SCKO DSCs were frequently located beneath the endocytotic pits of the plasma membrane in the absence of elongation (Fig. 6d, e, Table S1e and Video S3), suggesting that tubular invagination is a form of autophagic membrane remodeling; that is a tubular phagophore, in DSCs. There were no pre-SP clusters along the nuclear membrane of *Atg7*-SCKO DSCs (Fig. 6e), indicating the specificity of their localization.

In mRNA-Seq analysis, we found that 6 genes out of 10 genes related to endocytotic recycling, such as *Arf6,* were increased (Fig. 2c and Table S2). We also found an increase in the induction of Rab11a and Atg9b protein expression, both of which are associated with the interaction among recycling endosomes/plasma membrane and autophagy [36, 37], in injured sciatic nerves compared to uncut control nerves (Fig. 6f-h, Fig. S5f), potentially suggesting an alteration in the mechanism of phagophore membrane recruitment in DSCs.

### p62 mediated the selective affinity of the SP for myelin excretion in DSCs

**Fig. 7:**
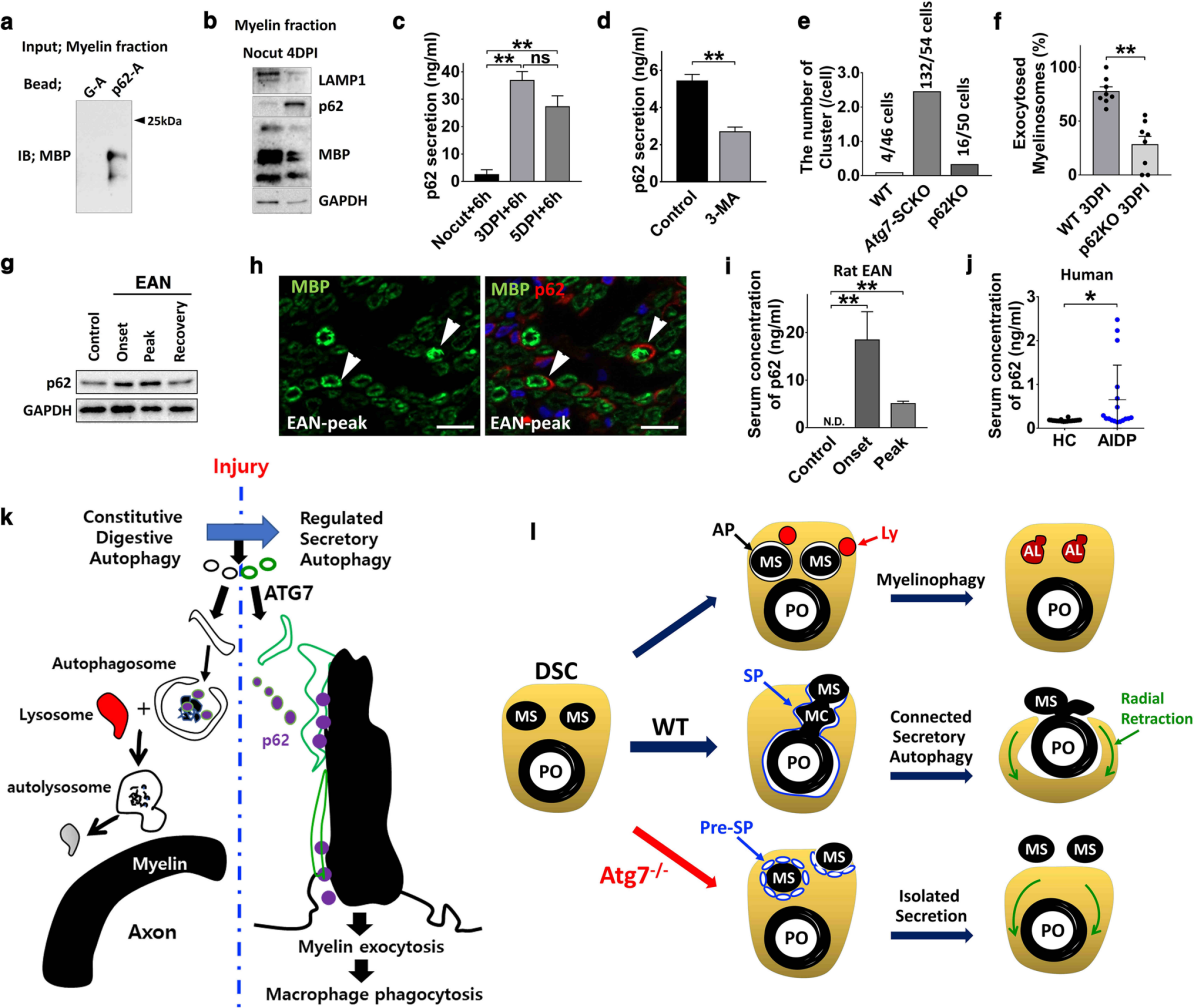
p62 mediated myelin excretion in peripheral demyelination. **a** Binding of recombinant UBA domain of p62 with myelin basic protein (MBP). G-A: glutathione-agarose bead, p62-A: p62-agarose bead. **b** Purified myelin fraction was immunoblotted with indicated antibodies. **c** ELISA results showing the secretion of p62 in the sciatic nerve explant culture medium. Nocut + 6 h: 6 h explant cultures with uncut nerves. 3 DPI + 6 h: 6 h explant culture with preinjured nerves at 3 days before the culture. **d** ELISA results showing the secretion of p62 in the medium of sciatic nerve explant culture for 3 days. 3MA: 3-methyladenine. **e** Quantification of the number of pre-SP clusters in DSCs. **f** Quantification of exocytosed myelinosomes in WT and p62 KO mice at 3DPI. Each dot indicates the percentage of exocytosed myelinosome in each DSC. **P < 0.01. **g** Sciatic nerve lysates from experimental autoimmune neuritis (EAN) rats were immunoblotted for the indicated antibodies. **h** Representative IF staining on p62 and MBP in EAN nerves. Arrow: SCs. Scale bar = 20 μm. **i, j** ELISA results showing p62 levels in the serum of rat EAN (**i**) and patients with acute inflammatory demyelinating polyradiculoneuropathy (AIDP, **j**). *HC* healthy controls. *P < 0.05, **P < 0.01, *ns* non-significant. Data are represented as mean ± standard error of mean. **k** Model of autophagy reprogramming from digestive to secretory autophagy for myelin clearance in SC after nerve injury. **l** Connected secretory autophagy model. *AP* autophagosome, *AL* autolysosome, *Ly* lysosome

Our findings indicated that *Atg7*-dependent generation of the SP contributes to demyelination via selective interaction of the SP with myelin in DSCs. Since autophagy receptors, such as p62, mediate the interaction of the phagophore with cargo and because p62 expression was increased in DSCs, we examined whether p62 can mediate the affinity of the SP for myelin in DSCs. According to a previous study, p62 interacts with myelin basic protein via its ubiquitin-associated domain [38]. We also observed an interaction between the recombinant p62 ubiquitin-associated domain of p62 and myelin basic protein in peripheral myelin (Fig. 7a). Moreover, we found high levels of p62 in purified myelin from the sciatic nerves at 4 DPI (Fig. 7b), suggesting the localization of p62 in degenerating myelin.

We next investigated whether p62 secretion from DSCs is induced during myelin exocytosis. Sciatic nerves were preaxotomized and three days later, sciatic nerve explants were cultured for 6 h, and p62 levels in the culture medium were analyzed by ELISA. The levels of p62 were higher in the medium of preinjured nerve explant cultures than in the medium of uncut sciatic nerve explant cultures (Fig. 7c). In addition, p62 was found at high levels in the medium of non-preinjured sciatic nerves cultured for 3 days, and the addition of 3-methyladenine (an autophagy inhibitor) into the culture medium strongly suppressed the secretion of p62 (Fig. 7d). Thus, p62 appears to be secreted from DSCs in an autophagy-dependent manner.

We also examined SBF-SEM images of DSCs from p62 KO mice at 3 DPI to determine whether the phagophore-mediated myelin clearance process was altered in p62 KO mice. First, we observed many uncompacted degenerating myelin in myelin fragmentation sites, while there were few pre-SP clusters in p62 KO DSCs (16 clusters in 50 DSCs from p62 KO mice, Fig. 7e and Video S6). In addition, 81.4% of 70 myelinosomes (78.7% in WT DSCs, *P* > 0.05) were still connected to the primary ovoid in DSCs from p62 KO mice in serial SEM images, suggesting that phagophore expansion was minimally affected by the loss of p62 in DSCs. In contrast, the percentage of exocytosed myelinosomes showed a significant decrease in p62 KO DSCs compared to WT DSCs (Fig. 7f), indicating the contribution of p62 to myelin exocytosis by DSCs.

SC transdifferentiation to DSCs has been recently suggested to be involved in inflammatory demyelinating neuropathies [33]. We thus examined whether a similar alteration in autophagy is induced during demyelination in EAN, a rat model of inflammatory demyelinating neuropathy [18]. The levels of p62 were found to be increased in the SCs of EAN rats compared to those of control rats (Fig. 7g, h). In addition, we investigated whether secreted p62 is found in the sera of EAN rats and human patients with acute inflammatory demyelinating polyneuropathy (AIDP). ELISA results showed that p62 was undetectable in the sera of control rats; however, 10–20 ng/ml p62 was observed in the sera of animals with EAN at the onset of the disease (Fig. 7i). Furthermore, a significant increase in p62 levels was observed in the sera of AIDP patients, but p62 was rarely detected in the sera of healthy controls (Fig. 7j).

## Discussion

### Myelin exocytosis with autolysosome inhibition

In the present study, we examined autophagic flux in SCs after nerve injury using an autophagy reporter mouse. We observed the suppression of both autophagic flux and active lysosomal maturation in adult DSCs following nerve injury; this finding is unexpected due to the potential autolysosomal digestion of myelin debris, called myelinophagy, in DSCs [12]. Even though high expression of lysosomal-associated membrane protein-1 has been considered an indicator of lysosome induction in SCs upon injury [11], recent studies have revealed that it is expressed in a wide spectrum of endocytic organelles, including late endosomes [39]. Considering the extensive plasma membrane remodeling required for myelin excretion by DSCs during WD, the dramatic induction of lysosomal-associated membrane protein-1 in DSCs might be related to lysosomal exocytosis for membrane repair, not solely to myelin digestion [40–42]. Along with the reduction in autophagic flux in DSCs, we observed the transcriptional downregulation of autolysosomal maturation in the mRNA-Seq analysis of the injured sciatic nerves and the accumulation of an autophagy substrate p62. Since we performed mRNA-Seq analysis before macrophages infiltrated in the injured nerve, and because SCs are the major population of the sciatic nerves, we consider that the altered autolysosomal genes might be principally derived from DSCs. Thus, our results lead to the novel proposal that SCs perform myelin clearance while inhibiting canonical digestive autophagy during WD. Recent studies have shown that a non-destructive type of autophagy, namely secretory autophagy, is tightly associated with the inhibition of autophagic flux [3, 6], and our serial ultrastructural analysis indicated that DSCs may utilize a form of secretory autophagy for myelin clearance during WD. In line with this, we observed autophagy-dependent secretion of p62 and high localization of p62 in the purified myelin fraction during peripheral demyelination. We found a reduction in the accumulated p62 at 6 days after injury compared to the level at 3 days after injury (and also in ex vivo), which may be due to the destruction of secreted p62 potentially by tissue proteases. Taken together, secretory autophagy implicated in p62-mediated myelin excretion may underlie a mechanism of myelin transfer, in particular the large myelin ovoid, from SCs to macrophages during demyelination. A recent report [33] showing a delay in myelin lipid digestion by macrophages in *Atg7*-SCKO mice further supports this hypothesis. Taken together, our findings indicate that a novel autophagy reprogramming from digestive autophagy to secretory autophagy underlies the mechanism of rapid myelin clearance in SCs after nerve injury (Fig. 7k).

However, our results do not completely exclude the possibility of myelinophagy in DSCs. Lutz et al. described the late-onset of myelin digestion by DSCs [13], suggesting that myelinophagy by DSCs may participate in demyelination to digest myelin remnants within DSCs after early exocytotic clearance of bulky myelin via secretory autophagy. Further studies comparing the contribution of myelinophagy and secretory autophagy in myelin clearance at different time points following injury will be required.

### “Connected secretory autophagy model” for myelin ovoid clearance in DSCs

Using SBF-SEM, the present study revealed for the first time the ultrastructural features of the phagophore-related myelin excretion process in WD. Myelin was exposed to the external environment of SCs via fusion of myelin enclosing the SP with the plasma membrane. Here, we propose a “connected secretory autophagy model” of rapid myelin exocytosis by DSCs (Fig. 7l). We found that most myelinosomes were connected to the primary myelin ovoid via the myelin chain and that the connection relied on membrane coverings derived from *Atg7*-dependent expanded phagophores around myelins. As shown in Fig. 7l, when the myelinosomes are not connected to the primary ovoid (as in *Atg7*-SCKO DSCs), the secretion of isolated myelinosomes would not lead to exposure of the intracellular primary ovoid; however, if a myelinosome is connected to the primary ovoid, the extracellular exposure of a part of the myelinosome may result in the simultaneous exocytosis of all connected myelins, including the primary ovoid. On the other hand, we observed Atg7-dependent membrane expansion not only around degenerating myelin (SP) but also from the plasma membrane (tubular phagopore). Since SPs and tubular phagophores are both *Atg7*-dependent phagophores, we hypothesized that the fusion of both types of phagophores may contribute to the connection of intracellular myelin to the plasma membrane for myelin exocytosis (Fig. S5g).

Although demyelination by DSCs was delayed in *Atg7*-SCKO mice, a large amount of demyelination still occurred in *Atg7*-SCKO mice [12, 13]. SBF-SEM revealed many excreted myelinosomes within the endoneurial tubes in *Atg7*-SCKO mice (~ 70% of WT DSCs), indicating the occurrence of *Atg7-*independent myelin exocytosis by DSCs. In *Atg7*-SCKO mice, pre-SP clusters surrounded myelin and accumulated beneath the plasma membrane, suggesting that pre-SPs might possess the critical properties of SPs (albeit a reduced efficiency), including affinity for myelin and ability to fuse with the plasma membrane, thereby enabling the exocytosis of isolated myelinosomes in *Atg7*-SCKO mice (Fig. 7l). However, since a large proportion of the connections between myelinosomes and the primary ovoids were lost in *Atg7*-SCKO mice (Fig. 4c), the exocytosis of the whole of the primary ovoid seemed to be delayed.

### Source of SPs

In the present study, we provided several pieces of evidence of the contribution of myelin lamellae to the formation of the SP in DSCs [17]. Since the ER and Golgi complex, the principal contributors to initial phagophore formation, are not highly concentrated in the myelin fragmentation sites away from the nucleus, degenerated myelin membranes in those areas might be the core platforms for not only the formation of the initiation membrane of autophagosomes but also for SP expansion. It was recently shown that Rab11a-dependent LC3 conjugation leads to autophagosome formation from recycling endosomes, which originate from endocytosis of the plasma membrane [36], and we found high levels of injury-induced Rab11a expression in myelin fragmentation sites in DSCs. In addition, regarding the induction of genes related to recycling endosomes in DSCs, the activation of the interaction of recycling endosomes and the autophagy pathway may be implicated in SP formation from the plasma membrane at those sites. Further studies to reveal the molecular mechanism of SP formation will provide important insight into the pathophysiology of demyelination.

Conclusively, our data showed that *Atg7*-dependent but autolysosome-independent myelin exocytosis by DSCs might be ideally suited to achieve rapid clearance of large bunches of myelin ovoids during WD. This process may be related to SC reprogramming to DSCs and repair cells following injury. Thus, our findings reveal an additional plastic change in SCs with the respect to autophagy process during demyelination.

## Supplementary Information

Below is the link to the electronic supplementary material.

Fig. S1 Autolysosome suppression in DSCs. Fig. S2 Transcriptional reprogramming in DSCs. Fig. S3 Representative EM images of myelin exocytosis in DSCs. Fig. S4 Pre-SPs in Atg7-SCKO DSC. Fig. S5 Phagophore generation from uncompacted degenerating myelin1 (DOCX 1665 KB)

Table S1 Analytical methods of SBF-SEM (DOCX 113 KB)

Table S2 Gene lists of GO term analysis (XLSX 369 KB)

Table S3 Lists of antibodies and primers (DOCX 26 KB)

Video S1 SBF-SEM images of WT DSC at 3 DPI. Red arrows indicate the connections of APPM and plasma membrane (MP4 2249 KB)

Video S2 Electron tomography of DSC of Atg7-SCKO mice. Red arrows indicate pre-SPs (MP4 2680 KB)

Video S3 SBF-SEM images of Atg7-SCKO DSC at 3 DPI. Red arrows indicate pre-SP clusters around myelin and under the plasma membrane (MP4 1902 KB)

Video S4 SBF-SEM images of WT DSC near the end of the primary ovoid at 3 DPI. Red arrows indicate pre-SP clusters (MP4 1797 KB)

Video S5 SBF-SEM images of Atg7-SCKO DSC near the end of the primary ovoid at 3 DPI. Red arrows indicate the connections of APPM and plasma membrane (MP4 1778 KB)

Video S6 SBF-SEM images of p62 KO DSC at 3 DPI. Red arrows indicate perimyelin membranes (MP4 1443 KB)

## Data Availability

Supplementary data to this article can be found online.
