# Supplementary material for: Adaptive autophagy reprogramming in Schwann cells during peripheral demyelination
Source: Cell Mol Life Sci. 2023 Jan 9;80(1):34. doi: 10.1007/s00018-022-04683-7 (PMC9829575; doi:10.1007/s00018-022-04683-7)
Supplement: Supplementary file 1 — Fig. S1 Autolysosome suppression in DSCs. Fig. S2 Transcriptional reprogramming in DSCs. Fig. S3 Representative EM images of myelin exocytosis in DSCs. Fig. S4 Pre-SPs in Atg7-SCKO DSC. Fig. S5 Phagophore generation from uncompacted degenerating myelin1 (DOCX 1665 KB) [file 18_2022_4683_MOESM1_ESM.docx]

**Adaptive autophagy reprogramming in Schwann cells during peripheral demyelination**

Young Rae Jo, Yuna Oh, Young Hee Kim, Yoon Kyung Shin, Hye Ran Kim, Hana Go, Jaekyoon Shin, Hye Ji Park, Hyongjong Koh, Jong Kuk Kim, Jung Eun Shin, Kyung Eun Lee, Hwan Tae Park

**Supplementary Figures and Legends**

**
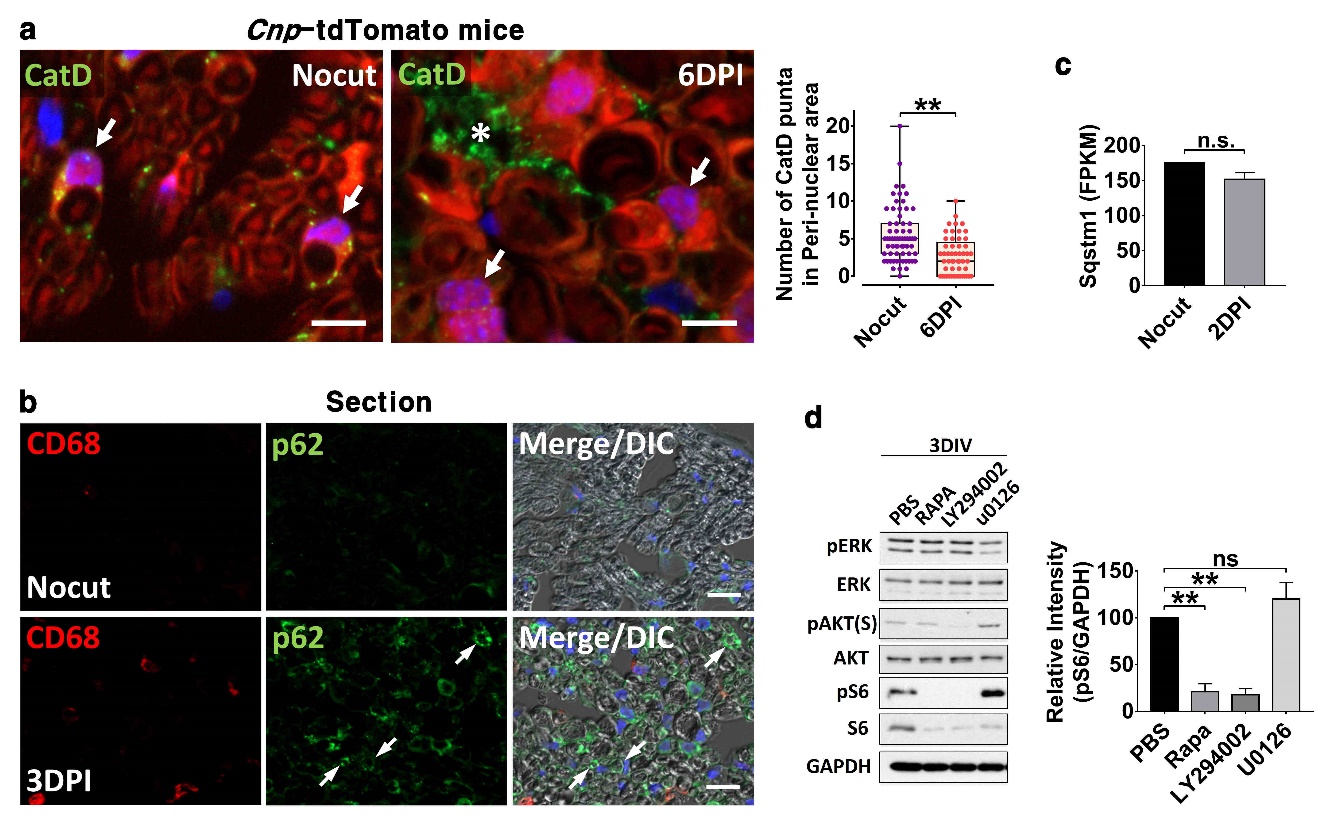
**

**Fig. S1** Autolysosome suppression in DSCs. **a** Immunofluorescent (IF) staining against cathepsin D (green puncta) in the sciatic nerve cross sections of *Cnp*-td-Tomato mice that represent Tomato fluorescence in SCs. Arrows; td-Tomato-positive SCs showing nucleus (red), Asterisk; cathepsin D-positive macrophage. Quantification of the number of perinuclear cathepsin D puncta in DSCs. **; *P* < 0.01. **b** Representative IF staining against p62 in the cross sections of the distal stump of the sciatic nerve after injury. Arrows; peri-myelin p62 stain. IF staining against CD68 was used for the identification of macrophage. DIC, differential interference contrast. Scale bar in **a** and **b** = 10 μm. **c** The results of mRNA-Seq analysis showing the value of p62 (Sqstm1) transcripts. FPKM, fragments per kilobase of transcript per million. **d** Western blot analysis showing the PI3K-mTORC1 axis in the sciatic nerve explant culture with quantification. RAPA; rapamycin. 3DIV; 3 days *in vitro*.


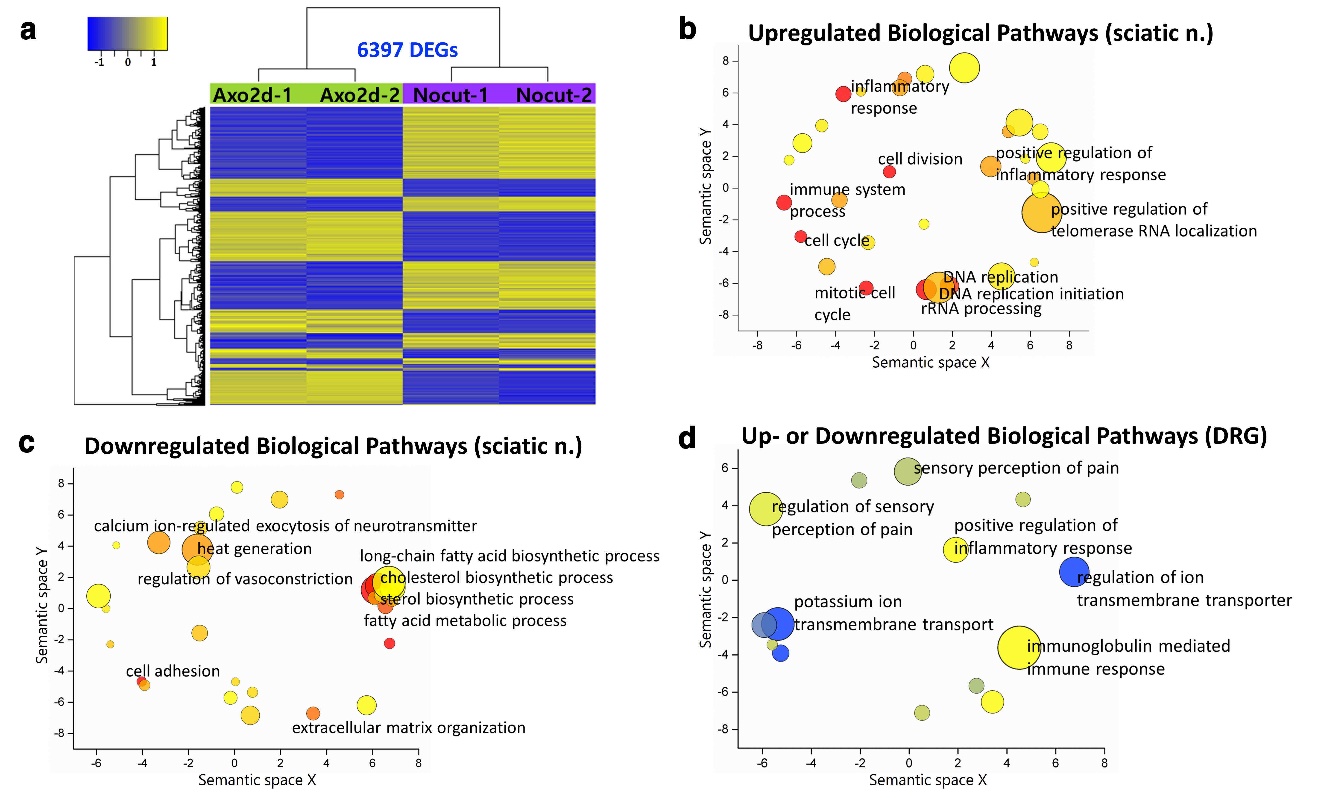


**Fig. S2** Transcriptional reprogramming in DSCs. **a** Heatmap analysis of differentially expressed genes (DEGs) in the sciatic nerves following axotomy (Axo). Duplicates of mRNA-Seq analysis. (6397 DEGs, FC > 2, raw *P* < 0.05). **b-d** Biological pathways (BP) of up **b** or downregulated **c** DEGs from DSCs identified by DAVID GO analysis were visualized using REVIGO. The circle diameter reflects the −log10-*P*-value and colors indicate the fold enrichment scores. Horizontal and vertical axes of the plot represent semantic space (SimRel vector space); the circles' closeness on the plot reflects their semantic similarity between the GO terms. **d** Biological pathways of DEGs from dorsal root ganglion (DRG) following nerve injury identified by DAVID GO analysis were visualized using REVIGO.


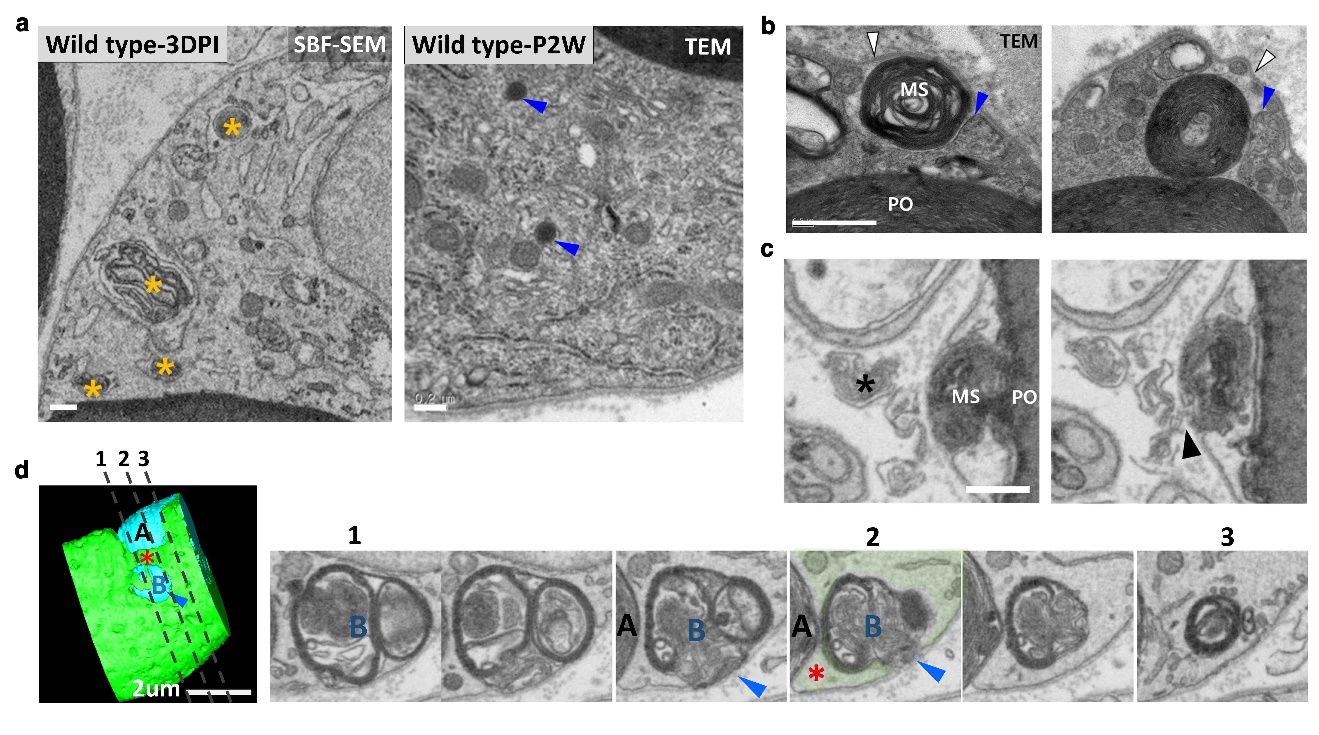


**Fig. S3** Representative EM images of myelin exocytosis in DSCs. **a** Left panel; SBF-SEM image of a DSC at 3 DPI showing numerous myelin enclosing vesicular structures (asterisks) without lysosomes. Right panel; TEM image showing lysosomes in the cytoplasm of SC at postnatal 2 weeks (P2W, arrowheads). Scale bar = 0.2 μm. **b** TEM images of exocytosed myelinosomes. PO; primary ovoid, white arrowheads; basal lamina, blue arrowheads; SC plasma membrane. **c** SBF-SEM images showing myelin secretion to the extracellular space (asterisk). **d** SBF-SEM images showing two exposed myelinosomes (A, B). Three dimensional reconstructions are the views of a DSC from different angles. Blue surfaces are exposed myelin and green areas are the SC plasma membrane. The numbers (1-3) indicate section planes. Arrowheads in drawings and SBF images indicate the same location. Asterisks; SC plasma membrane between exposed myelins.


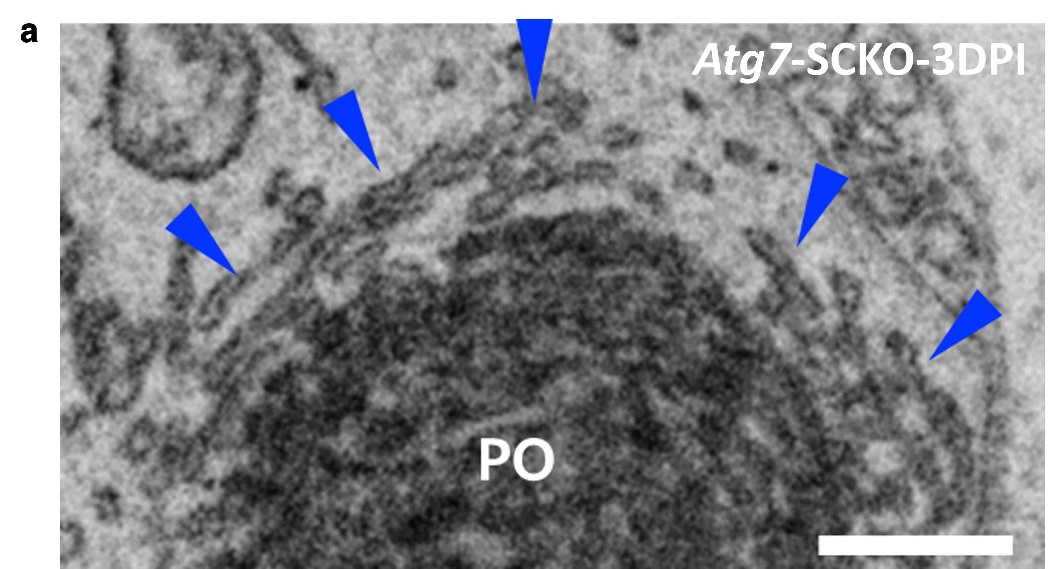


**Fig. S4** Pre-SPs in *Atg7*-SCKO DSC. **a** Representative SBF-SEM image showing multiple pre-SPs (arrowheads) around an end of a primary myelin ovoid in *Atg7*-SCKO DSC. Scale bar = 0.2 μm.


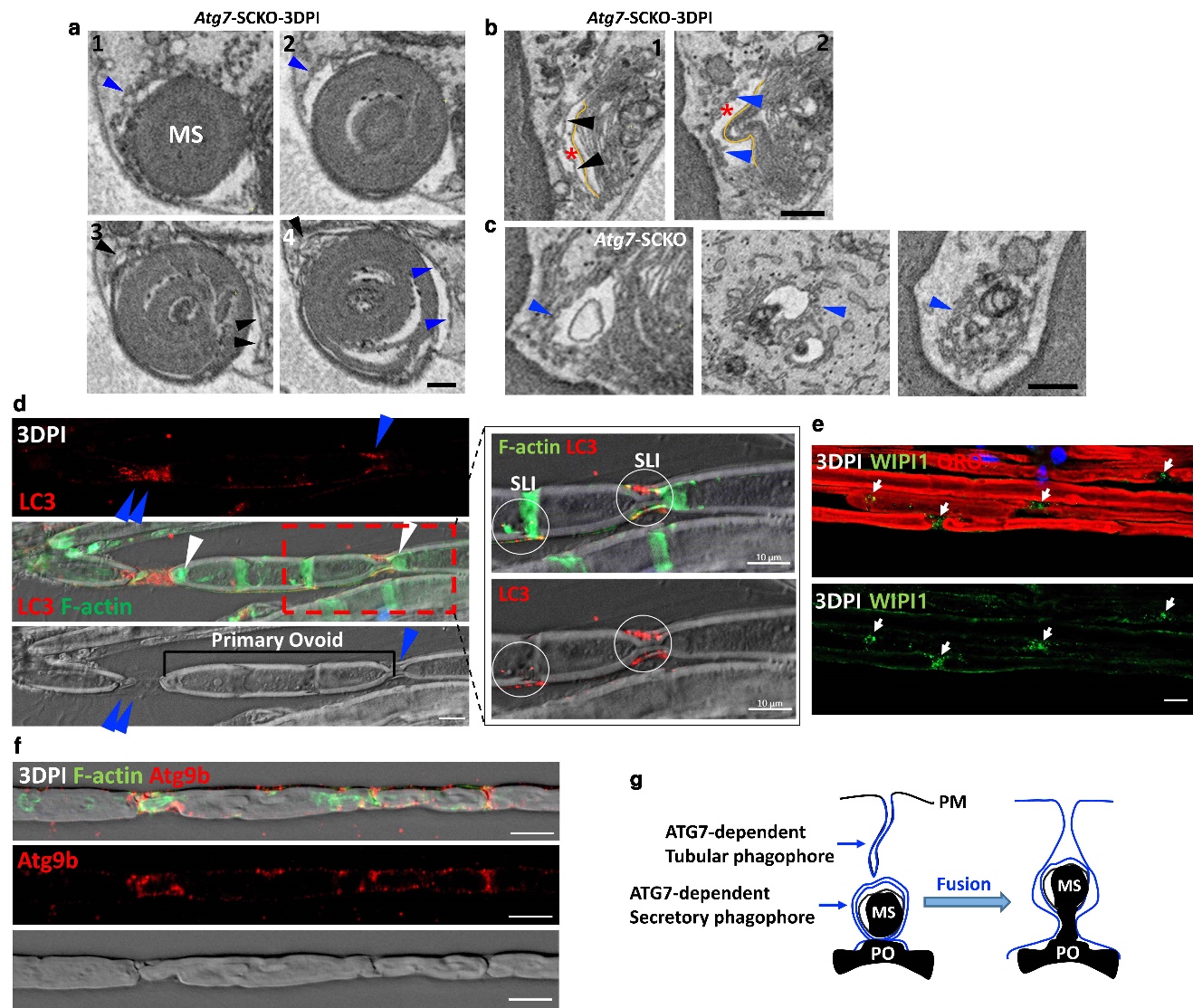


**Fig. S5** Phagophore generation from uncompacted degenerating myelin. **a, b** Serial SEM images showing a transition of a myelin lamella (black arrowheads) to multiple pre-SPs (blue arrowheads) in *Atg7*-SCKO DSC. **b** Orange lines (right figures) indicated the same membrane. Asterisk; the same space. **c** SBF-SEM images showing pre-SPs (blue arrowheads) surrounding single layer of myelin lamella (black arrowheads) in *Atg7*-SCKO mice at 3 DPI. Scale bar = 10 μm. **d** Representative images of LC3 and phalloidin actin staining in the teased nerve fibers of WT mice at 3 DPI. Blue arrowheads; myelin fragmentation sites near demolishing Schmidt-Lanterman incisure (SLI, white arrowheads). Red box; enlarged area. F-actin; phalloidin actin stain for actin filaments. **e** Representative IF images of WIPI1 staining (arrows) in the teased nerve fibers at 3 DPI. ORO; oil-red O stain for myelin. Scale bar = 10 μm. **f** Representative images of Atg9b and phalloidin actin staining in the teased nerve fibers of WT mice at 3 DPI. **g** Hypothetic drawing of myelin exocytosis via the fusion of *Atg7*-dependent tubular and secretory phagophores. PM; plasma membrane.
