# Supplementary material for: Adaptive autophagy reprogramming in Schwann cells during peripheral demyelination
Source: Cell Mol Life Sci. 2023 Jan 9;80(1):34. doi: 10.1007/s00018-022-04683-7 (PMC9829575; doi:10.1007/s00018-022-04683-7)
Supplement: Supplementary file 2 — Table S1 Analytical methods of SBF-SEM (DOCX 113 KB) [file 18_2022_4683_MOESM2_ESM.docx]

**Table S1**. Analytical methods of SBF-SEM

|  | SBF-SEM | Cartoon | Data | Object | Sample count and measurement | Expression |
| --- | --- | --- | --- | --- | --- | --- |
| **a** | 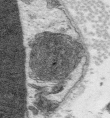 | 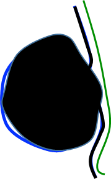 | **Exocytosed myelinosome**:  Myelinosome contact to the basal lamina (**Fig. 4f** and **7f**). | 70-126 isolated myelinosomes in a single section | Serial SEM images of 8 DSCs in each of WT, *Atg7*-SCKO, and p62 KO mice (400-1036 serial SEM sections in each cell) | Percentage of exocytosed myelinosome |
| **b** | 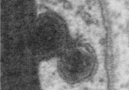 | 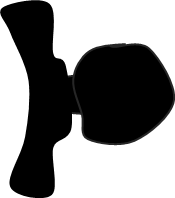 | **Connection of myelins:**  Myelinosome contact to the primary myelin ovoid. | 70-126 isolated myelinosomes in a single section | Serial SEM images of 8 DSCs in each of WT, *Atg7*-SCKO and p62 KO mice (400-1036 serial SEM sections in each cell) | Percentage of the myelinosome connected to the primary ovoid |
| **c** | 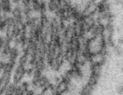 | 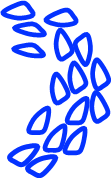 | **Pre-SP cluster**. | Pre-SP cluster consisted of more than 10 pre-SPs in a single section | Serial SEM images of 46-54 DSCs in each of WT, *Atg7*-SCKO and p62 KO mice (400-1036 serial SEM sections in each cell) | Number of cluster |
| **d** | 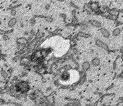 | 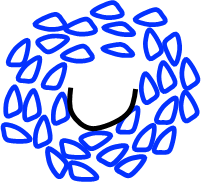 | Pre-SP encircling one or no myelin lamella (**Fig. 5**). | Pre-SP encircling less than one myelin lamella in a single section | Serial SEM images of 8 DSCs in each of WT and *Atg7*-SCKO mice (400-1036 serial SEM sections in each cell) | Number of cluster |
| **e** | 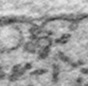 | 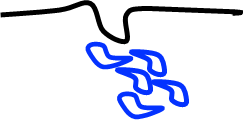 | Pre-SP under the endocytotic pit (**Fig. 6d**). | Pre-SP cluster consisted of more than 5 pre-SPs under endocytotic pit in a single section | Serial SEM images of 8 DSCs in each of WT and *Atg7*-SCKO mice (400-1036 serial SEM sections in each cell) | Number of cluster |
