# Supplementary material for: Adaptive autophagy reprogramming in Schwann cells during peripheral demyelination
Source: Cell Mol Life Sci. 2023 Jan 9;80(1):34. doi: 10.1007/s00018-022-04683-7 (PMC9829575; doi:10.1007/s00018-022-04683-7)
Supplement: Supplementary file 4 — Table S3 Lists of antibodies and primers (DOCX 26 KB) [file 18_2022_4683_MOESM4_ESM.docx]

**Table S3**

# List of antibodies

| **ANTIBODY** | **SOURCE** | **Catalog Number** |
| --- | --- | --- |
| ATG7 | Sigma-Aldrich | Cat#A2856 |
| SQSTM1/p62 | Abcam | Cat#ab109012 |
| WIPI1 | Abcam | Cat#ab128901 |
| β-actin | Santa Cruz | Cat#A5441 |
| NGFR p75 (C-20) | Santa Cruz | Cat#sc-6188 |
| c-Jun (H-79) | Santa Cruz | Cat#sc-1694 |
| Zero (C-19) | Santa Cruz | Cat#sc-18533 |
| Myelin Basic Protein | Milipore | Cat#AB980 |
| CD68 | Bio-Rad | Cat#MCA1957 |
| LAMP-1 (1D4B) | Santa Cruz | Cat#sc-19992 |
| Phospho-S6 (Ser235/236) | Cell Signaling Lab | Cat#4856 |
| S6 (5G10) | Cell Signaling Lab | Cat#2217 |
| Phospho-Akt (Ser473) | Cell Signaling Lab | Cat#4058 |
| Akt | Cell Signaling Lab | Cat#9272 |
| Phosphorylated ERK1/2 | Cell Signaling Lab | Cat#9101 |
| ERK1/2 | Cell Signaling Lab | Cat#9102 |
| Cathepsin D | R&D system | Cat#AF1029 |
| Rab11a | Thermo Fisher Scientific | Cat#71-5300 |
| Atg9b | Thermo Fisher Scientific | Cat#PA5-20998 |
| Anti-rabbit IgG, HRP-linked | Cell Signaling Lab | Cat#7074 |
| Anti-mouse IgG, HRP linked | Santa Cruz | Cat#sc-2005 |
| Anti-goat IgG, HRP linked | Santa Cruz | Cat#sc-2020 |
| Anti-rabbit IgG, Alexa Fluor 488 conjugated | Molecular Probes | Cat#A-21206 |
| Anti-Rabbit IgG, Cy3 conjugated | Millipore | Cat#AP182C |
| Anti-Goat IgG, Alexa Fluor 488 conjugated | Thermo Fisher Scientific | Cat#A-11055 |
| Anti-Goat IgG, Cy3 conjugated | Millipore | Cat#AP180C |
| GAPDH [EPR16884] | Abcam | Cat#ab181603 |
| LC3b antibody kit | Invitrogen | Cat#L10382 |

# List of primers

| **Oligonucleotides (Primer DNA)** | **SOURCE** | **Catalog Number** |
| --- | --- | --- |
| *Osblp1a* (Forward):  5'-GAAGTTCTGGGGCAAGAGTG-3' | Bioneer | N/A |
| *Osblp1a* (Reverse):  5'-TTTGTCCCCAGTCTTGTGG-3' | Bioneer | N/A |
| *Vamp-8* (Forward):  5'-ATCATCCCTGCGAGCCTATCCT-3' | Bioneer | N/A |
| *Vamp-8* (Reverse):  5'-GACCTTTTTTGGCTAAACGCTTTC-3' | Bioneer | N/A |
| *Borcs5* (Forward):  5'-ATCATCCCTGCGAGCCTATCCT-3' | Bioneer | N/A |
| *Borcs5* (Reverse):  5'-GACCTTTTTTGGCTAAACGCTTTC-3' | Bioneer | N/A |
